# Dietary Fibre and Chronic Kidney Disease: A Systematic Review of Effects on Inflammation, Uraemic Toxins, Nutritional Status, Kidney Function, and Gut–Liver–Kidney Axis Mechanisms

**DOI:** 10.3390/nu18091341

**Published:** 2026-04-24

**Authors:** Anna Gabriela Mojak, Monika Bronkowska

**Affiliations:** Institute of Health Sciences, University of Opole, 45-061 Opole, Poland; monika.bronkowska@uni.opole.pl

**Keywords:** chronic kidney disease, dietary fibre, soluble fibre, inflammation, uraemic toxins, nutritional status, kidney function, gut–liver–kidney axis, microbiota, Dietary Inflammatory Index, systematic review

## Abstract

Background: Dietary fibre has been increasingly recognised for its potential role in modulating inflammation, gut-derived uraemic toxins, nutritional status, and kidney-related outcomes in chronic kidney disease (CKD), particularly through mechanisms involving the gut–liver–kidney axis. While nutritional management in CKD has traditionally focused on protein intake, despite growing evidence supporting soluble and insoluble types, the role of dietary fibre remains insufficiently reflected in clinical guidelines. Objective: This systematic review evaluated the effects of dietary fibre intake on inflammatory markers, gut-derived uraemic toxins, nutritional status, kidney function, and mechanistic pathways relevant to gut–liver–kidney axis among CKD patients. Methods: PubMed, Scopus and Medline Complete were searched for observational and interventional human studies. Review articles and animal studies were excluded. A total of 45 met eligibility criteria. Risk-of-bias (RoB) was assessed using domain-based tools, and findings were synthesised narratively across predefined outcome domains. Results: Higher fibre intake was generally associated with reductions in interleukin-6 (IL-6) and selective improvements in inflammatory tone including Tumor Necrosis Factor alpha (TNF-α), while effects on *C*-reactive protein (CRP) varied. Several fermentable fibres were frequently linked with reduced gut-derived uraemic toxins, including indoxyl sulphate (IS), p-cresyl sulphate (pCS), and less consistently trimethylamine-N-oxide (TMAO). Nutritional markers such as albumin, BMI and overall diet quality were typically maintained or improved. Kidney function was stable across short-term interventions, with suggestions of slower decline in longer studies incorporating fibre-rich dietary patterns. Mechanistic studies frequently reported increased saccharolytic activity and favourable changes in fermentation profiles. Despite growing evidence, soluble fibre remains an underrepresented component in CKD dietary guidelines, warranting further high-quality interventional studies to confirm its therapeutic potential.

## 1. Introduction

### 1.1. Chronic Kidney Disease (CKD)

Chronic kidney disease (CKD) is a progressive and incurable condition affecting about 9–16% of the global population [1]. It is defined by an estimated glomerular filtration rate (eGFR) of less than 60 mL/min/1,73 m^2^ and a urinary albumin-to-creatinine ratio (UACR) of 30 mg/g or higher. The prevalence is particularly high in countries such as India and China [2]. Alarmingly, approximately 15% of individuals in the United States are affected [2], where the Western diet, characterised by high intake of animal protein and low fibre is widespread. Even when patients survive the early stages of CKD, the disease often progresses to end-stage kidney disease (ESKD), which requires renal replacement therapy, including dialysis or transplantation [3]. These interventions significantly reduce quality of life for both patients and their families. Dialysis, in particular, carries a high mortality risk, with approximately 10% of patients dying within the first 90 days and 20% within the first year of treatment [3,4].

It is well established in the literature that dietary fibre intake is associated with a lower risk of diabetes mellitus (DM), cardiovascular disease (CVD), and cancer, but its effects in CKD remain unclear. Despite the growing burden of CKD, current nutritional strategies have largely focused on protein intake, while the potential role of dietary fibre in slowing disease progression remains underexplored. This review aims to synthesise existing evidence on dietary fibre interventions, particularly simple modifications applicable in early-stage CKD, and highlight their potential clinical relevance.

### 1.2. Microbiota and Gut–Kidney–Liver Axis

The interaction between the gut microbiota and CKD is a significant area of research, highlighting the bidirectional relationship known as the “gut–kidney axis”. This relationship impacts the progression of CKD and overall patient health. Dysbiosis is consistently associated with CKD progression [5]. The intestine, connected to the liver via the portal vein system, serves as a reservoir of microorganisms and a source of metabolites that regulate intestinal function and modulate the activity of both the liver and kidneys [6]. A disrupted intestinal barrier exposes the liver to the translocation of food metabolites, bacteria, fragments of their DNA and their metabolites such as lipopolysaccharide (LPS), as well as toxic molecules [6]. These factors activate the hepatic innate immune system, resulting in the production of trimethylamine-N-oxide (TMAO) and reduced production of short-chain fatty acids (SCFAs) [6]. To maintain homeostasis, the liver stimulates pro-inflammatory, antiviral and apoptotic pathways. Cytokines such as tumour necrosis factor (TNF-α), interleukin 1β (IL-1β), and interleukin 6 (IL-6) are secreted [6]. These stimulate the liver to secrete acute-phase proteins such as amyloid A, lipopolysaccharide binding protein (LBP), fibrinogen (factor I of the plasma coagulation cascade), *C*-reactive protein (CRP), and ceruloplasmin, which counteract liver damage [6]. Dysbiosis, which compromises the integrity of the intestinal barrier and leads to translocation and production of uraemic toxins, mediates systemic inflammation through the liver, which is also associated with chronic kidney disease [6]. Toxic effects primarily impact the liver and kidneys [6]. More fat accumulating around the liver drives systemic inflammation through the secretion of pro-inflammatory cytokines (TNF-α, IL-1β and IL-6). This inflammatory state further disrupts the intestinal barrier and accelerates CKD progression [6]. This condition is associated with increased visceral obesity, hypertension, and insulin resistance [6]. Moreover, marked disturbances in lipid metabolism are observed [6]. These include a disrupted lipid profile, altered lipid oxidation, elevated triglycerides (TGs), increased low-density lipoprotein (LDL) levels transporting cholesterol to tissues from the liver, and decreased high-density lipoprotein (HDL) levels transporting cholesterol from tissues to the liver [6].

### 1.3. The Role of Fibre in CKD Progression

Dietary fibre plays a significant role in managing and potentially slowing the progression of CKD. Its mechanisms of action include modulation of the gut microbiota from a more proteolytic to a more saccharolytic profile, reduction in uraemic toxins and lowering systemic inflammation. Dietary fibre also influences the composition and metabolism of the gut microbiome, which is crucial for reducing uraemic toxin production and systemic inflammation. This modulation helps preserve kidney function and slow CKD progression [7,8,9,10]. Fibre intake can decrease levels of gut-derived uraemic toxins such as indoxyl sulphate (IS) and p-cresyl sulphate (pCS), which are linked to CKD progression [9,10,11]. High-fibre diets are associated with reduced systemic inflammation, with improvements noted in markers such as CRP, IL-6, and TNF-α [11,12,13]. Clinical benefits include improved markers of renal health, cardiovascular health and nutritional balance. Fibre supplementation has been shown to lower serum urea and creatinine levels, which are classical markers of renal health [14]. In CKD patients, fermentable fibres such as inulin may slow the progression of cardiovascular complications, including aortic and cardiac calcification, and reduce left ventricular mass index and fibrosis [10]. Fibre also helps manage other CKD-related complications such as obesity, diabetes, and dyslipidaemia by promoting a healthier gut microbiota and supporting metabolic regulation [8,15]. Both fermentable and non-fermentable fibres have important roles. Additionally, insoluble fibre accelerates intestinal peristalsis, shortening the transit time and reducing the risk of potassium absorption and subsequent hyperkalaemia. Despite these benefits, many CKD patients consume less than the recommended amount of dietary fibre [15]. Fermentable fibres like inulin are particularly effective in reducing uraemic toxins and improving gut health [10,13]. Diets rich in fibre and low in animal proteins, such as the Mediterranean diet, are recommended, as they support CKD management and reduce cardiovascular risks [16,17].

#### 1.3.1. Dietary Index for Gut Microbiota

The Dietary Index for Gut Microbiota (DI-GM) is a validated tool designed to assess the impact of diet on gut microbiota diversity. It was developed through a systematic review of 106 studies examining the relationship between dietary components and gut microbiota composition in adults [18]. The index includes 14 dietary components: 10 beneficial components—fermented dairy, whole grains, dietary fibre, soybean, broccoli, avocados, cranberries, chickpeas, coffee and green tea—and four unfavourable components—red meat, processed meats, refined grains, and high-fat diets (≥40% of energy from fat). Each component is scored as follows: beneficial foods receive 1 point if intake is above the sex-specific median and 0 if below. Unfavourable foods receive 1 point if intake is below the median or below the fat threshold and 0 if above. The total score ranges from 0 to 14, with higher scores indicating a diet more supportive of gut microbiota diversity.

#### 1.3.2. Dietary Inflammatory Index

The Dietary Inflammatory Index (DII) is a literature-derived, population-based scoring system designed to assess the inflammatory potential of an individual’s diet based on the intake of up to 45 food parameters and their effects on six key inflammatory biomarkers: IL-1β, IL-4, IL-6, IL-10, TNF-α, and CRP [19]. An overview of Dietary Inflammatory Index (DII) score categories and their dietary interpretation is provided in Table 1.

The DII includes up to 45 dietary parameters, such as nutrients, flavonoids, and food items. Each component is assigned an inflammatory effect score based on evidence from the literature. Individual intake is standardised against a global reference database. The final score reflects the overall inflammatory potential of the diet: positive scores indicate a pro-inflammatory diet, whereas negative scores indicate an anti-inflammatory diet.

#### 1.3.3. Adapted Dietary Inflammatory Index (ADII)

The Adapted Dietary Inflammatory Index (ADII) is a modified version of the original Dietary Inflammatory Index (DII), designed to quantify the inflammatory potential of an individual’s diet based on their intake of specific nutrients and food components. It retains the core methodology of the original DII, which links dietary components to inflammatory biomarkers such as CRP, IL-6, and TNF-α. It includes 22 dietary components, tailored to the corresponding food-composition database. It excludes certain nutrients and food items (notably ethanol, total fat and energy) to avoid inflated estimates of inflammatory potential. Pro-inflammatory nutrients (e.g., saturated fats and cholesterol) increase the ADII score, while anti-inflammatory nutrients (e.g., fibre, *n*-3 polyunsaturated fatty acids PUFAs, and vitamins) decrease the ADII score.

#### 1.3.4. Protein to Fibre Ratio (P/F)

The protein-to-fibre intake ratio (P/F) is a dietary metric used to evaluate the balance between protein and fibre consumption in an individual’s diet. It has attracted attention for its role in shaping the gut microbiome, influencing metabolic health, and potentially reducing the risk of chronic diseases such as CVD and CKD. It is calculated as

$$protein-to-fibreratio=\frac{daily\;protein\;intake\;[g]}{daily\;fibre\;intake\;[g]}$$

The ratio reflects dietary balance more accurately than considering protein or fibre alone. Nutrients are not consumed in isolation. A high ratio indicates a diet high in protein but low in fibre, which may suggest high intake of animal products and low intake of fruits, vegetables, legumes and whole grains. In CKD and CVD research, a higher P/F ratio has been associated with increased inflammation, greater production of uraemic toxins, and higher risk of cardiovascular events.

High P/F ratios (i.e., high protein and low fibre) may promote proteolytic fermentation in the colon, leading to the production of toxic metabolites associated with inflammation and disease. Low P/F ratios (i.e., balanced or high fibre relative to protein) support saccharolytic fermentation, which benefits gut health and helps control systemic inflammation.

### 1.4. The Aim of the Review

This systematic review provides an updated and thematically structured synthesis of evidence on the effects of dietary fibre intake on inflammatory markers, gut-derived uraemic toxins, nutritional status, kidney function, and mechanistic pathways in the gut–liver–kidney axis among patients with chronic kidney disease (CKD).

While previous reviews have examined the effects of dietary fibre on inflammation and uraemic toxins in CKD, they often focused solely on randomised trials or lacked mechanistic insights. This review addresses a broader clinical and biological context by integrating observational and interventional human studies, excluding animal models, and exploring the gut–liver–kidney axis as a key pathway.

## 2. Materials and Methods

### 2.1. Search Strategy

This systematic review was conducted in accordance with the PRISMA 2020 [20] and PRISMA-S [21] reporting guidelines, following methodological guidelines from the Cochrane Handbook for Systematic Reviews of Interventions [22]. Internal validity of included studies was assessed using RoB 2.0 (for RCT), ROBINS-I (for non-randomised and observational studies), the Newcastle–Ottawa Scale (for cohort studies), and the JBI Critical Appraisal Tool (for cross-sectional studies).

A structured literature search was performed in PubMed, Scopus, and Medline Complete to identify human studies evaluating dietary fibre intake or fibre-based prebiotic interventions in CKD. The search covered the period from January 2006 to December 2025. Search terms combined controlled vocabulary (MeSH) and free-text expressions relating to CKD (e.g., fibre, fiber, dietary fibre/fiber, prebiotic, resistant starch, inulin, β-glucan, and psyllium). Across the databases, the search retrieved a total of 4517 records. Full reproducible search strategies, including Boolean operators, interfaces, and search dates, are reported in the Supplementary Material (PRISMA-S checklist). Language (English) and human-study restrictions were applied during screening. Attempts were also made to search ClinicalTrials.gov and the Cochrane Central Register of Controlled Trials. However, technical limitations prevented the reliable export of records. As preregistered trials typically contribute limited outcome data for nutrition-focused systematic reviews, these sources were not included in the final screening set. This decision is documented in the Limitations section.

### 2.2. Records Management

A total of 4467 records were retrieved across the three databases. All records were imported into Rayyan (rayyan.ai) for screening and reference management because of import inconsistencies encountered in Mendeley. Duplicate detection followed a two-step procedure. First, automatic deduplication based on identical DOIs was performed in Rayyan. Second, remaining potential duplicates were manually verified by comparing titles, authors, publication years, journal names, and DOIs, as discrepancies in metadata formatting across PubMed, Scopus, and Medline Complete occasionally prevented automated matching.

In total, 2070 duplicate records were removed, leaving 2397 unique records for title and abstract screening. Screening of titles and abstracts was performed entirely within Rayyan. Of the 2397 screened records, 118 full-text articles were retrieved for eligibility assessment.

**Figure 1:**
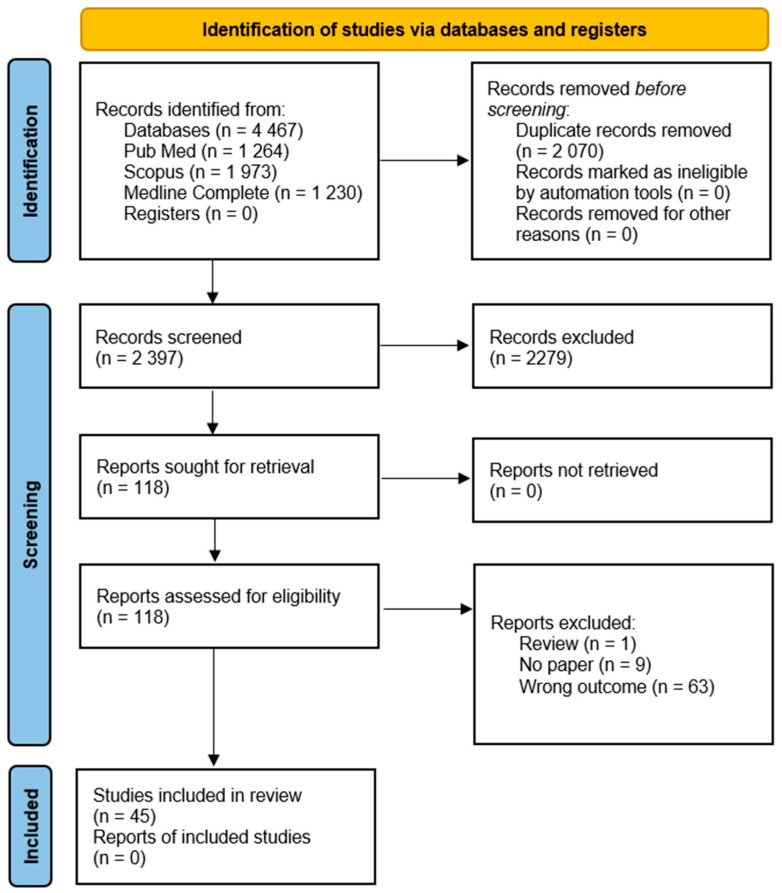
Flow diagram of the study selection process.

Full-text articles corresponding to the 118 records identified through title and abstract screening in Rayyan were retrieved manually using institutional and open-access sources and imported into Mendeley via the browser plugin. Mendeley was used exclusively for full-text reading, annotation and highlighting, as well as for citation management. After full-text review, 45 studies met the inclusion criteria and were incorporated into the qualitative synthesis. The study selection process is summarised in Figure 1.

All screening steps (title/abstract and full-text assessment) were performed by one reviewer (A.M.) and independently checked by a second reviewer (M.B.). Any discrepancies were resolved by discussion. Rayyan was used to support screening and automated DOI-based deduplication, without applying machine-learning-based automatic inclusion decisions.

### 2.3. Selection Criteria

#### 2.3.1. Inclusion Criteria

Studies were included if they met the following criteria:

- Original observational (cross-sectional, cohort, or case–control) or interventional (RCT, non-randomised trials) design;
- Conducted exclusively on human subjects,
- Only studies published in English were considered;
- Assessed dietary fibre intake in relation to CKD outcomes;
- Reported outcomes related to:
  ○ Kidney function (serum creatinine, eGFR, and albuminuria);
  ○ Inflammation (CRP, IL-6, and TNF-α);
  ○ Uraemic toxins (IS, pCS, indole-3-acetic acid (IAA), p-cresyl glucuronide (pCG), and TMAO);
  ○ Nutritional status, quality of life, or mortality,
- Provided quantitative data (e.g., odds ratio (OR), hazard ratio (HR), β, and *p*-values).

#### 2.3.2. Exclusion Criteria

Studies were excluded if they met any of the following criteria:

○ Were review articles;
○ Were conducted on animals;
○ Evaluated probiotics, synbiotics, postbiotics, or mixed interventions in which dietary fibre was not the primary component;
○ Did not specifically address both CKD and dietary fibre.

### 2.4. Data Extraction

Data from eligible full-text articles were extracted into a structured Excel matrix designed for this review. The matrix captured detailed information on study identification (DOI and reference), study design and sample characteristics, CKD stage or dialysis modality, intervention or exposure type and dose, comparator groups, duration, data-collection methods and authors’ stated objectives and key findings. In addition to these study-level variables, all predefined outcome domains were extracted, including kidney function indicators, gut-derived uraemic toxins, inflammatory markers, nutritional status measures and gut–liver–kidney axis mechanisms. Numerical results were extracted whenever reported. The same Excel matrix was also used to perform full risk-of-bias (RoB) assessment for each study, with item-level signalling questions and overall domain judgements.

All available results compatible with the predefined outcome domains were sought, regardless of measurement method, statistical model or time point. When multiple results were reported, primary outcomes (as defined by study authors) or the most commonly reported measures were extracted. Data extraction was performed by one reviewer (A.M.) and checked for accuracy by a second reviewer (M.B.).

### 2.5. Synthesis Methods

A narrative synthesis approach was applied, following the principles of SWiM (Synthesis Without Meta-Analysis). Studies were grouped according to predefined outcome domains: inflammatory markers, gut-derived uraemic toxins, kidney function, nutritional indicators, and gut–kidney axis mechanisms.

Meta-analysis was not performed due to substantial methodological and statistical heterogeneity across studies, including differences in study design, fibre type and dose, exposure, analytical methods, and non-comparable effect measures. Findings were therefore interpreted narratively, focusing on the direction and consistency of effects and methodological rigour. Effect measures were extracted and presented as reported in the original studies, including absolute concentrations, mean or median changes from baseline, between-group differences, and model-derived estimates such as regression coefficients, odds ratios or hazard ratios. For inflammatory markers and gut-derived uraemic toxins, absolute and percentage change values were additionally calculated from reported numerical data to allow consistent comparison across studies. No transformation or standardisation of effect measures was applied, and no pooled effect estimates were generated. Results of individual studies were tabulated in five structured summary tables aligned with the predefined outcome domains, using a standardised format to facilitate comparisons across studies.

### 2.6. Additional Information

This review was conducted using Rayyan web-based tool only for literature screening and automated deduplication. The review was registered in PROSPERO on 31 December 2025 (CRD420251275865). No amendments were made to the PROSPERO-registered protocol. Microsoft 365 Copilot (Microsoft Corp., Redmond, WA, USA; web-based service, accessed December 2025; GPT-5-based language model) was used solely for translation and language refinement.

Template data extraction forms, extracted data, analytical datasets and analytical code were not made publicly available, as the review did not generate reusable computational code or de-identified datasets. All materials used in the review are described within Methods. No additional materials are available. Supplementary Material, including PRISMA documentation, the PROSPERO record and full RoB assessments were provided to the editors and reviewers as part of the peer-review process.

## 3. Results

### 3.1. Dietary Fibre and Inflammatory Markers

Across observational and interventional studies, higher dietary fibre intake or supplementation with fermentable fibre was generally associated with modest improvements in systemic inflammation, although the magnitude and consistency varied by fibre type, study design, and outcome domain. Observational evidence from large renal cohorts (GCKD, ULSAM, and PIVUS) showed inverse associations between habitual fibre intake and circulating CRP, despite substantial confounding and serious RoB arising from self-reported dietary intake and cross-sectional design limitations [23,24,25,26]. A population-based study from Iran suggested that the association may differ by smoking status. Higher fruit and vegetable intake as a proxy for dietary fibre was associated with lower hs-CRP only among smokers [27]. Given the variability in study design, fibre type and inflammatory endpoints, Table 2 presents a standardised summary of results to inform the narrative interpretation.

#### 3.1.1. Haemodialysis Populations—Inflammation

Randomised trials in haemodialysis populations demonstrated a more coherent pattern for individual inflammatory pathways. Supplementation with resistant starch (HAM-RS2) repeatedly reduced IL-6 and TNF-α, with supportive reductions in oxidative stress markers such as MDA across several trials [29,31,32], including one study [32] that reported clear improvements in IL-6, TNF-α, and MDA over 8 weeks of treatment [32]. CRP concentrations, however, remained broadly unchanged across HAM-RS2 interventions [32,33], suggesting that fermentable starches may modulate cytokine-driven inflammatory signalling rather than acute-phase responses.

One RCT assessed a multiplex panel of chemokines and growth factors rather than classical systemic markers [30]. RS significantly reduced RANTES (CCL5), IP-10 (CXCL10), and PDGF-BB, although no data on CRP, IL-6, TNF-α, or other prespecified inflammatory markers were collected. RANTES/CCL5 is a pro-inflammatory chemokine implicated in leukocyte recruitment and atherosclerotic processes, IP-10/CXCL10 attracts activated T-cells via CXCR3, and PDGF-BB promotes chemoattractant and mitogenic activity related to vascular remodelling. These reductions indicate immunological modulation, but their clinical interpretation is limited because no systemic inflammatory markers, uraemic toxins, microbiota endpoint, or nutritional indices were assessed. In addition, a larger randomised placebo-controlled study of water-soluble fermentable fibre in HD patients reported broad improvements across inflammatory and oxidative stress pathways, with marked falls in hs-CRP, IL-6 and IL-8, together with clear reductions in MDA and substantial increases in total antioxidant capacity [13]. TNF-α also declined within the intervention arms, although between-group differences were not statistically significant, and the open-label design warrants cautious interpretation. The placebo group showed no meaningful change, suggesting a biological signal despite methodological limitations [13].

#### 3.1.2. Predialysis Populations—Inflammation

In predialysis CKD, inulin supplementation produced modest reductions in IL-6 [39], although effects on hs-CRP and TNFα remained neutral. One RCT in predialysis CKD patients reported no changes in hs-CRP, IL-6, TNF-α, IL-10 or MDA over 16 weeks of RS supplementation [38]. A more recent trial in CKD stages 3–4 evaluating a Korean Mediterranean high-fibre diet found no significant between-diet differences for multiple inflammatory cytokines, with only a non-significant within-period reduction in granulocyte-macrophage colony-stimulating factor (GM-CSF) [42]. Cohort evidence reported attenuated rises in IL-6 and CRP among higher-fibre groups over 18 months, although strong potential for residual confounding and reverse causation limits interpretability [44]. Across the four protein–fibre strata: (low-protein low-fibre, low-protein high-fibre, high-protein high-fibre, and high-protein high-fibre), IL-6 and CRP increased across all groups. IL-6 showed a graded fibre-related pattern, with higher-fibre strata (LPHF and HPHF) demonstrating smaller increases than their lower-fibre counterparts. CRP rose across all combinations, with more variability between groups but without evidence of attenuation or reduction in any subgroup [44]. A separate trial using a low-protein diet (LPD) with added inulin reported no notable change in IL-6 or IL-1β, while small decreases were observed in CRP, TNF-α and NOX2 [43]. In an earlier RCT with high RoB evaluating Gum Arabic at 10–40 g/day for 4 weeks, CRP decreased modestly, although IL-6 and TNF-α remained unchanged [41]. In the most recent RCT with serious RoB evaluating p-inulin in predialysis CKD, plasma inflammatory biomarkers (CRP, IL-6, and TNF-α) did not change significantly [40]. Complementary cross-sectional data at serious RoB comparing LPHF with HPLF dietary patterns in CKD 3–4 similarly demonstrated selective inflammatory differences, with lower IL-18 and monocyte chemoattractant protein-1 (MCP-1) in the higher-fibre subgroup while other cytokines remained unchanged, indicating attenuation of specific inflammatory pathways rather than broad cytokine suppression [46].

Overall, inflammation worsened in every group, although IL-6 tended to rise more slowly where fibre intake was higher, while CRP remained heterogeneous and did not show a clear fibre-dependent pattern [44]. Complementary cross-sectional evidence from NHANES III showed that, for each 10 g/day higher fibre intake, adults with CKD had substantially lower odds of elevated CRP. Total fibre was associated with 38% lower odds (OR = 0.62), insoluble fibre with 45% lower odds (OR = 0.55), and soluble fibre with 74% lower odds (OR = 0.26), indicating a strong inverse relationship between habitual fibre intake and systemic inflammation in CKD [45].

#### 3.1.3. Peritoneal Dialysis Populations—Inflammation

Not all fibre-based interventions produced detectable inflammatory effects. A crossover RCT in PD patients [37] reported no changes in hs-CRP, IL-6, IL-10, TNF-α or LPS following RS intake delivered via unripe banana flour, likely influenced by limited adherence and difficulties achieving the target dose. Similarly, a subsequent small trial with p-inulin showed that only plasma-soluble CD14 (sCD14) declined significantly, with concentrations lower at week 16 than at week 8, whereas IL-6, hs-CRP and TNF-α exhibited no significant changes [36]. Another RCT in PD patients using a mixed soluble fibre formulation showed a reduction in IL-8 and favourable shift in IL-18 relative to placebo, with no significant effects on IL-6, TNF-α, IFN-ɣ or IL-1β [34]. A short, randomised Mediterranean diet counselling intervention in PD patients similarly produced no measurable effects on IL-6 or TMAO over four weeks, despite improvements in diet adherence [35].

Overall, effects were most frequently observed for IL-6 reductions, particularly in resistant-starch and inulin trials, while CRP responses remained variable. Several small, short-duration trials with either some concerns or serious RoB, combined with reliance on observational designs for large cohorts, restrict the certainty with which causal inferences can be drawn. Reductions in chemokines reported in de Paiva 2020 [30] should be interpreted as exploratory rather than equivalent indicators of changes in systemic inflammation. Higher dietary fibre intake and fermentable fibre supplementation showed downward trends in key inflammatory markers, especially IL-6, whereas CRP effects remained heterogenous. Benefits appeared strongest in haemodialysis RCTs using HAM-RS2 and inulin-based interventions, although the overall certainty of evidence remains low to moderate, due to small sample sizes, short treatment periods, and RoB concerns across the available literature. Short counselling-based Mediterranean diet intervention similarly produced neutral IL-6 and TMAO responses over four weeks [35].

### 3.2. Dietary Fibre and Uraemic Toxins

Across interventional and observational studies fibre-based interventions produced variable toxin-lowering effects, with reductions most frequently observed for pCS, IS, and pCG, while responses differed by dialysis modality, toxin fraction, fibre type and study design [29,38,39,47,48,49]. Findings tended to be clearer in low-risk trials and more heterogeneous in those at some concerns or serious RoB. Given the variability in study design, fibre type and uraemic toxin endpoints, Table 3 presents a standardised summary of results to inform the narrative interpretation.

#### 3.2.1. Haemodialysis Populations—Uraemic Toxins

Across HD RCTs, reductions in uraemic toxins were selective, with effects most frequently observed for pCS. A longer HAM-RS2 intervention reduced pCS (~23%) with IS unchanged [38]. Shorter RS2 studies showed a decrease in IS [29] or p-cresol [33], while other trials found no change in IAA or related solutes [28]. Observational data indicated lower IS and pCS with higher fibre intake and more favourable P/F ratios, whereas constipation phenotypes were associated with substantially higher pCS across serum and urinary fractions [49,55]. Across HD studies, results tended to be clearer in low-risk trials, whereas studies at some concerns or high RoB showed more variable toxin responses.

#### 3.2.2. Peritoneal Dialysis Populations—Uraemic Toxins

Most PD studies reported no measurable reductions in circulating uraemic toxins. Soluble-fibre dietary mixtures (SDFs) and RS banana flour produced no changes in pCS, IS, IAA or TMAO [34,37]. Inulin-type fructans prevented increases in faecal indole, but serum toxins and urinary/dialysate removal remained unchanged [50]. Weekly measurements in a non-randomised crossover study demonstrated significant overall time effects for IS, pCS and TMAO [36]. Visual inspection of the trajectories suggested that levels rose during the p-inulin periods and declined towards post-intervention values, although numerical concentrations were not reported [36]. This study demonstrated the feasibility and biological potential of prebiotics to modulate host-microbial co-metabolism in PD patients. However, interpretation is limited by a serious overall RoB [36]. Another non-randomised trial with serious RoB reported no effects of p-inulin on IS or pCS [40]. Overall, PD interventions did not show measurable toxin-lowering effects. Patterns were broadly similar across RoB categories, with predominantly null findings regardless of trial quality.

#### 3.2.3. Predialysis Populations—Uraemic Toxins

In NDD-CKD, effects varied by fibre type and toxin fraction. Pea hull with inulin lowered serum p-cresol [53]. Inulin along with diet lowered both IS and pCS [39], and β-glucan produced substantial reductions in free IS, free pCS and free pCG [47]. Arabinoxylan oligosaccharides from wheat bran (AXOS) did not reduce protein-bound toxins [48], while short β-glucan supplementation lowered TMAO without affecting IS or pCS [52]. FOS supplementation produced a non-significant trend towards lower serum total pCS [51]. Cross-sectional data in CKD 3–4 comparing low-protein high-fibre with high-protein low-fibre patterns similarly showed lower circulating TMAO in the higher-fibre subgroup, while IS and pCS remained unchanged, indicating selective modulation of gut-derived metabolites rather than uniform toxin lowering [46]. Cross-sectional data complement this pattern by showing that constipation itself—particularly the hard-stool phenotype (BSS < 3)—is associated with substantially higher total, free and urinary pCS, indicating that impaired gut motility may independently amplify microbial toxin generation in NDD-CKD [55].

Observational studies in adults showed inverse associations between fibre intake and toxin concentrations [49,54], though longitudinal patterns differed with diet composition [44]. Paediatric analyses reported reductions in free toxin fractions with higher fibre intake, while total fractions often remained unchanged [56,57]. Across NDD-CKD studies, reductions were more consistent in low-risk trials, whereas observational datasets at serious RoB showed greater heterogeneity. Overall free toxin fractions (free IS, free pCS, and free pCG) showed the most consistent responsiveness to fermentable fibre across non-dialysis populations.

### 3.3. Dietary Fibre and Nutritional Status

Across interventional and observational studies, dietary fibre intake and fermentable fibre supplementation generally maintained nutritional status in adults with CKD, with no indication of protein-energy wasting and occasional improvements in selected biochemical markers. Effects were broadly similar across dialysis modalities, with more consistent patterns in low-risk trials and greater heterogeneity in studies at some concerns or serious RoB. Given the variability in study design, fibre type and nutritional status endpoints, Table 4 presents a standardised summary of results to inform the narrative interpretation.

#### 3.3.1. Haemodialysis Populations—Nutritional Status

Across HD trials, fibre supplementation did not adversely affect nutritional markers, with stable or improved albumin, haemoglobin, BMI and dietary intake. Trials using RS reported maintained albumin, weight, BMI and haemoglobin [28,29,33], and all these studies were assessed at low to some concern of RoB.

A large dietary-education RCT demonstrated increases in albumin, haemoglobin and total protein alongside improvements in dietary quality [58]. This study was judged at some concerns for RoB. Soluble-fibre supplementation also increased haemoglobin, serum iron and ferritin [59], with the trial at high RoB.

Findings from non-randomised studies were more variable. Gum Arabic produced notable increases in albumin and total protein, but this evidence came from a study at critical RoB [60]. Overall, HD evidence indicates that fibre supplementation is nutritionally safe, with several trials showing improvements in albumin, haemoglobin and dietary adequacy.

#### 3.3.2. Peritoneal Dialysis Populations—Nutritional Status

In PD populations, dietary fibre interventions were generally neutral to modestly beneficial. RS flour and mixed soluble-fibre formulations maintained albumin, BMI, and protein/energy intake without nutritional harm [34,37]. These trials ranged from low to some concern of RoB. Fructo-oligosaccharides preserved albumin and weight and improved constipation scores [61], with the study judged at some concerns. Overall, fibre supplementation in PD preserved nutritional status without impairing albumin or protein and energy intake.

#### 3.3.3. Predialysis Populations—Nutritional Status

Across predialysis CKD, nutritional effects were consistently neutral or favourable. Inulin along with diet preserved albumin, prealbumin and transferrin [39], and β-glucan supplementation maintained body weight, BMI and albumin [52]. AXOS produced neutral effects on lipids, glucose, and anthropometry [48]. Lactulose preserved haemoglobin and albumin [62].

Observational studies consistently showed insufficient fibre intake, frequently below recommended ranges [64,65]. Low energy intake, reduced fruit and vegetable intake and excess protein intake were common, particularly in advanced CKD among socioeconomically deprived populations [66,67]. Longitudinal data indicated that higher fibre intake did not impair albumin, prealbumin or anthropometry [44]. Paediatric data were limited but showed no nutritional harm [56,57]. Overall, across NDD-CKD, fibre and prebiotic supplementation were nutritionally safe, with no evidence of weight loss, protein-energy wasting or deterioration in albumin or prealbumin.

### 3.4. Dietary Fibre and Kidney Function

Across interventional and observational studies, dietary fibre intake and fermentable fibre supplementation generally showed no clear evidence of short-term deterioration in kidney function. Most trials reported stable eGFR, creatinine, urea and BUN during the intervention periods, although study durations were generally brief and sample sizes small. Effects were more uniform in low-risk trials, whereas studies at some concerns or serious or critical RoB showed more variability [28,29,33,34,39,44,48]. Given the variability in study design, fibre type and kidney function endpoints, Table 5 presents a standardised summary of results to inform the narrative interpretation.

#### 3.4.1. Haemodialysis Populations—Kidney Function

Among HD populations, fibre supplementation did not adversely affect kidney function markers, with stable or unchanged eGFR, creatinine and urea across most RS2 interventions. Short-term RS2 trials reported no effect on creatinine or urea [28,29,38,68], and a longer 16-week RS2 intervention similarly produced no detectable change in kidney function markers [38]. Occasional reductions in creatinine or uric acid appeared in individual trials, though these effects were small or non-significant [33].

Non-randomised evidence was more variable, with Gum Arabic producing declines in urea, creatinine and uric acid in a critical-risk pre–post design [60]. Overall, HD studies did not show evidence of deterioration in kidney function, with no indication of harm from fibre supplementation.

#### 3.4.2. Peritoneal Dialysis Populations—Kidney Function

Across PD trials, findings were largely neutral. Inulin-type fructans, FOS and mixed soluble-fibre formulations did not affect residual GFR, creatinine clearance, cystatin C, ultrafiltration or eGFR [34,50,61]. Small reductions in eGFR or residual renal function reported in isolated studies did not exceed expected variability and were not statistically significant. Across PD studies, fibre supplementation was not associated with changes in renal function markers.

#### 3.4.3. Predialysis Populations—Kidney Function

In predialysis CKD, most RCTs showed stable kidney function, with inulin, β-glucan, AXOS and lactulose all demonstrating neutral effects on eGFR, creatinine and urea [39,48,52,62]. Observational data suggested that higher habitual fibre intake may be linked to slower eGFR decline, with >25 g/day showing more favourable trajectories over 18-month follow-up [44]. A 12-month Gum Arabic intervention also indicated possible stabilisation of eGFR, though evidence came from a study at serious RoB [63]. A crossover trial of a high-fibre Mediterranean diet reported stable eGFR compared with a small decline on a control diet [42]. A six-month dietary intervention combining an LPD with inulin likewise showed no meaningful change in eGFR but was accompanied by a reduction in serum uric acid and an improvement in bicarbonate concentrations in this very small high-risk study [43]. Across NDD-CKD studies, most interventions were renal-neutral, with isolated signals of slower decline in longer or whole-diet interventions. Consistent with these findings, a recent cross-sectional analysis at serious RoB in CKD 3–4 comparing low-protein high-fibre with high-protein low-fibre patterns likewise observed no differences in eGFR or creatinine between subgroups, reinforcing the overall pattern of short-term renal neutrality [46].

Across most short-term RCTs, kidney function markers were largely unchanged, while longer-duration or whole-diet interventions suggested possible benefit. However, certainty remains limited due to short follow-up and heterogeneity. Fibre-based therapies appear safe for kidney function, with longer-duration or whole-diet interventions indicating potential renoprotective effects, whereas short-term trials generally reported neutral outcomes. Overall certainty remains constrained by brief study durations and RoB concerns in several designs.

### 3.5. Gut–Liver–Kidney Axis Mechanisms

Across CKD populations and dialysis modalities, fermentable dietary fibre and prebiotic supplementation frequently reported modest shifts in gut microbial activity, gut-derived metabolites and stool-related functional markers relevant to the gut–liver–kidney axis. Effects varied across fibre types, dose and intervention duration, with patterns generally more uniform in low-risk trials and more variable in studies at some concerns or serious RoB [31,47]. Given the variability in study design, fibre type and gut–liver–kidney axis endpoints, Table 6 presents a standardised summary of results to inform the narrative interpretation.

#### 3.5.1. Haemodialysis Populations—Gut–Liver–Kidney Axis Mechanisms

Across HD trials, fermentable fibres frequently increased saccharolytic, SCFA-related taxa. RS2 increased the abundance of *Faecalibacterium* and showed upward trends in *Prevotella* and other saccharolytic genera [31]. Β-glucan and mixed soluble-fibre interventions increased circulating SCFAs and promoted a higher abundance of Lactobacillus, *Lactobacillaceae* and *Bifidobacterium adolescentis* [59], while lactulose increased Bifidobacteria and Lactobacilli [62]. A small high-RoB RCT with RS2 for 4 weeks also reported upregulation of several SCFA-producing taxa including *Roseburia*, *Ruminococcus gauvreauii* and *Oscillospiraceae*, alongside reductions in pro-inflammatory genera such as *Dialister*, although the short duration limits interpretability [68].

Markers of gut function also improved: increased bowel movement frequency was reported after RS2 [32] and dietary-education interventions including fibre expansion improved constipation patterns [58].

Overall, HD evidence indicated microbiota-directed shifts towards saccharolytic fermentation, accompanied by improvements in stool frequency and SCFA profiles, though sequencing depth and analytical methods varied across trials.

#### 3.5.2. Peritoneal Dialysis Populations—Gut–Liver–Kidney Axis Mechanisms

Among PD interventions, the soluble fibre mix did not alter α- or β-diversity but changed microbiota composition. Increases in *Prevotellaceae*, *Klebsiella*, *Veillonella*, *Paraprevotella*, *Lachnospiraceae UCG-010* and *Cetobacterium* versus placebo were observed, alongside within-group increases in *Clostridia*, *Oscillospirales* and *Cetobacterium.* Total SCFAs showed a non-significant upward trend, while propionate concentrations increased significantly after SDF [34]. Inulin-type fructans reduced *Bactroides thetaiotaomicron*, lowered faecal pH and limited increases in indole-producing taxa [50]. Functional outcomes also improved. FOS reduced colonic transit time, improved stool form and increased stool frequency [61]. RS-rich banana flour did not meaningfully change LPS or intestinal permeability markers [37]. Pea hull fibre improved stool frequency and lowered total cholesterol in CKD 3–5 patients [69]. In a metagenomic PD trial, p-inulin did not alter alpha-diversity and overall microbiota structure remained strongly driven by patient identity, although 86 bacterial strains changed significantly with treatment. PD patients also differed from healthy controls by 14 enriched taxa, compared with five enriched taxa in controls [36]. Across PD trials, fibre supplementation produced targeted microbial and fermentation-related changes, with generally improved stool transit and stability of microbial profiles, although interpretability remains limited by short intervention periods and heterogeneous analytical methods.

#### 3.5.3. Predialysis Populations—Gut–Liver–Kidney Axis Mechanisms

In predialysis CKD, β-glucan produced significant shifts in overall microbiome composition with greater temporal stability than control diets [47], a pattern suggestive of a transition towards saccharolytic community structure. Inulin-type fructans modified fermentation profiles and lowered faecal pH [50]. NHANES-based studies and paediatric cohorts showed that higher fibre intake was associated with lower levels of toxin precursors [25,56,57]. Constipation phenotypes were linked to higher levels of toxin-producing pathways. Individuals with slower transit exhibited markedly higher pCS levels in cross-sectional analysis [55]. Another p-inulin trial with serious RoB additionally reported significant shifts in microbial diversity, both alpha and beta, and genera-level composition, including increases in *Bifidobacterium, Anaerostipes, Lachnispira, Moryella, Negativibacillus, Ruminococcaceae,* and *Erysipelotrichaceae* [40]. In a longer dietary intervention in CKD 3–4, an LPD diet alone increased *Akkermansiaceae* and *Bacteroidaceae* and lowered *Christensenellaceae*, *Clostridiaceae*, *Lactobacillaceae*, and *Pasteurellaceae,* while the addition of inulin produced further increases in *Bifidobacteriaceae* and a reduction in *Enterobacteriaceae*, aligning with a shift towards a more saccharolytic profile in this very small, high-risk study [43]. Complementary cross-sectional evidence in CKD 3–4 comparing LPHF with HPLF patterns similarly demonstrated greater abundance of SCFA-producing taxa and depletion of proteolytic genera such as *Klebsiella* and *Clostridium sensu stricto 1*, consistent with the attenuation of dysbiosis-related gut–liver–kidney axis disturbances without indications of restored eubiosis [46]. Across NDD-CKD stages, findings supported modest, fibre-driven shifts in microbial ecology and fermentation patterns, with more pronounced effects seen in longer, higher-dose interventions.

Across all CKD stages fermentable fibre interventions generally modulated gut microbial composition, fermentation end-products and stool-related functional markers, with recurrent increases in SCFA-producing taxa and improvements in bowel transit. Effects were strongest in interventions delivering higher fermentable fibre doses or longer exposure periods, while short trials produced smaller changes.

## 4. Discussion

This systematic review indicates that fermentable dietary fibres appear to confer selective rather than uniform benefits in CKD, with their effects depending on fibre type, such as inulin, β-glucan, HAM-RS2 (RS2), and AXOS, the toxin fraction (free vs. total), the dialysis modality, and the exposure length. When interpreting findings across outcome domains, studies with low RoB were given greater interpretative weight, whereas results from studies with serious or critical RoB were considered exploratory. Given the substantial heterogeneity in fibre formulations, dosing strategies, population characteristics and outcome assessment, findings were synthesised narratively with emphasis on the direction rather than magnitude of effects. These overall patterns are reflected in individual studies, as illustrated by the following examples.

For inflammation, the most reproducible signal is a reduction in IL-6. In haemodialysis, RS2 administered at 16–25 g/day for 4–8 weeks reduced IL-6, and sometimes TNF-α, while attenuating oxidative stress. In contrast, CRP generally remained unchanged, suggesting a preferential modulation of cytokine pathways rather than acute-phase responses [29,31,32]. This pattern aligns with findings from a meta-analysis, which reported that prebiotics appeared most effective for IL-6 and TNF-α in dialysis populations, whereas reductions in CRP were observed mainly with synbiotic interventions [70]. Predialysis evidence accords with inulin 10 g/day for 12 weeks lowering IL-6 with neutral CRP or TNF-α [39] and short PD exposures (e.g., 30-day soluble-fibre mixes) showing limited cytokine change [34,37]. A longer predialysis intervention using an LPD with added inulin showed no change in IL-6, while modest reductions were observed in CRP, TNF-α and NOX2 in this very small, high-risk study [43]. A recent RCT meta-analysis across CKD further supports modest IL-6 and TNF-α reductions with fibre [11].

Overall, the evidence suggests that increasing dietary fibre intake or providing fibre-based supplements may beneficially modulate selected gut-derived uraemic toxins, certain inflammatory markers and aspects of gut–liver–kidney axis function in individuals with CKD. Effects on kidney function parameters and nutritional status indicators were generally small or absent across the short and heterogeneous interventions included in this review, which aligns with findings from prior narrative reviews and small-scale randomised trials reporting variable changes in creatinine, eGFR or albumin over weeks-long interventions. The pattern of results in domain-specific improvements in pCS, IS fractions or IL-6 in some but not all studies mirrors the broader literature suggesting that fibre-related effects may depend on dose, fermentability, baseline diet, microbiota composition and CKD stage. Signals tended to emerge with ≥8–12 weeks of exposure and with fermentable fibres at doses commonly around 10 g/day (inulin) or 16–25 g/day (RS2), while shorter courses were less reliable. Collectively, the available evidence supports a potential but not yet definitive role for dietary fibre as an adjunctive strategy targeting metabolic and inflammatory disturbances in CKD, while highlighting the need for larger, longer and more rigorously standardised trials [4,45,71,72]. An isolated 4-week RCT of Gum Arabic suggested a modest CRP reduction despite no change in IL-6 or TNF-α, which does not materially alter the overall interpretation of preferential IL-6 modulation with largely neutral CRP due to methodological limitations [41]. In HD, supplementation with 75% fermentable soluble fibre has produced broad improvements across several inflammatory and oxidative stress pathways without adverse effects on nutritional status, although important concerns in randomisation, blinding and analytical handling indicate that these findings should be interpreted cautiously and not considered definitive. In addition, the same intervention was associated with favourable shifts in metabolic profile, including improvements in lipid parameters, further supporting a potential yet still uncertain role of fermentable fibre in cardiometabolic modulation [13].

For gut-derived uraemic toxins, effects are fraction-specific and fibre-specific. The most robust reductions occur in free IS, pCS and pCG with inulin 10 g/day over 12 weeks and a 13.5 g blend containing ~3 g β-glucan over 14 weeks [39,47]. RS2 behaves more selectively: a 23% decrease in pCS at 16 weeks with 15–33 g/day occurred with IS unchanged [38], while several 4–8-week HD trials showed a decrease in IS with pCS unchanged or p-cresol decreases with IS unchanged [29,33]. AXOS 10 g/day for 4 weeks did not lower protein-bound toxins despite a small TMAO fall [48]. β-glucan 3 g/day in a non-randomised study decreased median TMAO ~17%with IS/pCS unchanged [52]. A recent RCT meta-analysis reported pooled reductions in IS and pCS after ≥4-week fibre interventions [11]. In PD, ITF 10 g/day (12 weeks) curtailed rises in faecal indole and lowered faecal pH but left serum IS and pCS unchanged, and a 15 g/day soluble fibre mix (30 days) did not alter toxins [34,50]. RS-rich banana flour (5–10 g RS/day) was neutral [37]. Evidence regarding the aryl hydrocarbon receptor (AhR) in human CKD is limited. In the only RCT assessing this pathway [28], resistant starch did not alter IAA concentrations or AhR mRNA expression, although a baseline correlation between IAA and AhR was observed. Given the absence of an intervention effect, AhR-related mechanisms cannot be inferred from the available data and are not interpreted further. Observational data were directionally aligned in that in haemodialysis, a lower P/F index and higher fibre intake were associated with lower IS/pCS—especially in anuric patients—while constipation relates to substantially higher pCS across fractions [45,49,54,55]. The P/F index in PD patients shifted slightly in favour of fibre but not significantly, and no correlations were observed between diet change and uraemic toxin levels, suggesting that microbiota-driven toxin modulation may require longer intervention [49].

For nutritional status, fermentable fibre was consistently safe across modalities: albumin, body weight, BMI and energy and protein intake were maintained in RS2, inulin, ITF, AXOS and mixed soluble-fibre interventions, including dialysis. Several trials reported improvements in albumin, haemoglobin or diet quality (e.g., high-fibre dietary education in HD, SDF with iron indices and SCFAs). In PD, a recent counselling-based Mediterranean diet intervention showed that 95% of patients consumed insufficient energy, one-third had inadequate protein intake, 62% were overweight or obese, and one-third met malnutrition criteria by a low phase angle, with no improvement in nutritional adequacy after the intervention, although potassium and phosphate remained stable [35]. These patterns reflect broader population-restriction practices. In a multicentre cohort from the Fukuoka Kidney Disease Registry (n = 4476), intake of vegetables, fruits and dietary fibre fell progressively from G1 to G5, with only 23% meeting vegetable recommendations and just 14% in G5. This supports the need to balance potassium safety with maintaining adequate fibre intake [66]. These conclusions mirror renal nutrition reviews noting habitually low fibre intake in CKD and endorsing food-first increases balanced against potassium safety [15]. Observational syntheses also suggest lower mortality and CVD events with higher fibre intake in CKD cohorts, though effect sizes attenuate with extensive adjustment in newer long-term datasets—supporting cautious interpretation when moving from biomarkers to outcomes [73,74,75]. Across studies, gastrointestinal tolerability was generally acceptable but inconsistently reported. Future trials should use standardised assessment tools alongside biochemical endpoints.

For kidney function, short-to-medium RCTs were largely neutral in the short-term, with eGFR, creatinine, urea and BUN unchanged across inulin, β-glucan, AXOS, RS2 and ITF, including a 16-week RS2 trial in CKD 3a-4 [38,39,48,50]. Longer-duration or whole-diet interventions suggested possible slower decline, with a Mediterranean high-fibre pattern showing stable eGFR relative to a small decline on control in a crossover RCT in CKD 3–4 [42] and long-term cardiometabolic trials showing a smaller decline on Mediterranean patterns, particularly in higher-risk subgroups [72]. These findings are consistent with the view that whole-diet, longer exposure rather than short, isolated supplements is required to influence renal trajectories. Hard clinical endpoints (ESKD, hospitalisation, and mortality) remain largely untested in fibre-specific RCTs and should be incorporated into longer, adequately powered designs.

Mechanistic and gut–kidney–axis signals suggest a shift towards more saccharolytic fermentation. Increases in SCFA-linked taxa (*Faecalibacterium*, *Lactobacillus*, and *Bifidobacterium*), lower faecal pH, improved transit, and, in some studies, higher circulating SCFAs have been reported. A pilot multi-omics work with p-inulin showed microbiome–metabolome changes compatible with increased saccharolysis, supporting biological plausibility [40]. A recent clinical review summarised the same mechanistic framework and called for harmonised microbiome endpoints in CKD trials [4]. The constipation–toxin association (higher pCS with slower transit) is repeatedly observed in CKD and supports integrating bowel-habit management within toxin-minimising care [4,55,76]. These mechanistic interpretations should therefore be viewed as exploratory and hypothesis-generating rather than definitive. Taken together, the evidence suggests a nuanced but favourable role of fermentable fibre in CKD with signals of IL-6 reduction (alongside largely unchanged CRP), lowering of free IS, pCS and pCG particularly with inulin or β-glucan, and pCS-selective responses with longer RS2 exposure. Across studies, interventions appeared nutritionally well-tolerated across dialysis modalities, and while renal parameters were generally stable in the short-term, longer observational or whole-diet comparisons have indicated a possible slower decline in kidney function over time. In contexts where toxin-related outcomes are being explored, research has pointed to stronger responses with certain fermentable fibres (e.g., inulin and β-glucan) and concurrent management of constipation. Dietary patterns characterised by lower P/F ratios have also been associated with more favourable toxin profiles, and some studies emphasise the value of assessing free toxin fractions as more responsive endpoints [11,45,49,54].

Key uncertainties remain: heterogeneity in fibre class, dose, duration, small samples and short exposures; diverse toxin and cytokine assays, including free and total fractions; and limited, non-uniform microbiome profiling. Future trials should prioritise head-to-head randomised comparisons of inulin, β-glucan and RS2 with ≥12–24-week exposure and harmonised free-fraction assays, integration of microbiome–metabolome endpoints, and pragmatic designs addressing potassium restriction, adherence and socioeconomic barriers. These needs are particularly acute in PD, where short exposures have generally yielded neutral findings.

Across observational studies included in this review, findings on clinical outcomes also aligned directionally with the biomarker patterns reported. A large prospective cohort of adults without CKD at baseline (110,412 participants, with 3507 incident cases) reported a clear inverse association between dietary fibre density and CKD risk, with progressively lower hazard ratios across fibre-intake quartiles and an approximate 3% reduction in CKD incidence per 1 g/1000 kcal higher fibre intake, in line with evidence across soluble and insoluble fibre [77].

Several cohorts also indicated that inadequate fibre intake frequently co-occurred with poor diet quality, suboptimal energy intake, and inflammation, creating challenges for separating associations from confounding [67]. In addition, socioeconomic disparities appear to modify these relationships. In a cross-sectional analysis from the HANDLS cohort, low adherence to a fibre-rich DASH dietary pattern was associated with markedly higher CKD prevalence, but only among individuals living in poverty. Those in the lowest DASH adherence tertile had over threefold higher odds of CKD compared with the highest tertile (OR = 3.15), whereas no association was observed in the nonpoverty group. Individuals experiencing poverty also had significantly lower fibre density (5.6 vs. 6.6 g/100 kcal), suggesting that social disadvantage may amplify the impact of poor diet quality on kidney health [67]. A cross-sectional analysis from the RenMex consortium (n = 2098) also showed that higher DII scores were associated with lower eGFR, with those in the highest tertile having substantially greater odds of mildly or moderately reduced kidney function compared with the lowest tertile [78]. A cross-sectional analysis from the Teheran Lipid and Glucose study (n = 4564) reported no isolated association between fibre and CKD but showed that higher dietary PRAL (Potential Renal Acid Load) was associated with lower eGFR and greater odds of CKD, with individuals in the most acid-producing diet quartile demonstrating significantly higher risk after multivariable adjustment (OR = 1.42) [79]. A Mediterranean cohort analysis from the PREDIMED trial baseline (n = 2123) similarly reported lower CKD prevalence with higher fibre intake, with the highest consumption quartile showing approximately one-third lower odds of CKD compared with the lowest. No association was observed with albuminuria, and higher *n*-6 PUFA intake was linked to reduced eGFR, indicating that broader dietary composition may influence fibre-related signals [80]. Recent NHANES analyses further highlight the relevance of fibre-related diet-quality metrics for CKD risk. In a nationally representative sample of 28 512 adults including 2951 CKD cases, higher fibre intake, operationalised via DI-GM, was independently associated with lower CKD prevalence (OR = 0.83) and markedly lower odds of very-high-risk CKD (OR = 0.58) [81]. A parallel NHANES dataset (n = 28,843 with 5 461 CKD cases) reported a 17.9% lower odds of CKD for higher fibre intake (OR = 0.82) [18]. Taken together, these findings provide directionally consistent cross-sectional associations linking fibre-rich dietary patterns with lower CKD prevalence across multiple U.S. datasets.

Higher dietary fibre intake has also been associated with favourable long-term outcomes in CKD cohorts. In the Chronic Renal Insufficiency Cohort, lower dietary fibre intake was associated with a higher risk of all-cause mortality, with participants in the middle tertile demonstrating a 19% higher mortality risk (HR = 1.19) and those in the lowest tertile an 11% higher risk (HR = 1.11) compared with the highest intake group [74]. In haemodialysis populations, higher fibre intake was likewise associated with lower cardiovascular mortality, with patients in higher-fibre tertiles exhibiting substantially reduced CVD mortality risk compared with those consuming <0.13 g/kg/day [73]. These findings accord with a large retrospective cohort of 742 maintenance haemodialysis patients in which those in the highest quartile of cereal-derived fibre intake had approximately 42% lower all-cause mortality (HR = 0.58) and approximately 47% lower cardiovascular mortality (HR = 0.53) [82]. In a prospective cohort study based on ULSAM, a higher P/F ratio was significantly associated with an increased risk of cardiovascular events in elderly men with CKD. For every increase in the P/F ratio, the risk of having a heart-related event increased by 33%, even after adjusting for other health and lifestyle factors (HR = 1.33) [26]. Across studies, patterns were more stable in low-risk analyses, whereas findings from cohorts at some concerns, serious or critical RoB, showed greater variability.

Additional evidence from a large NHANES-based cohort of 11 302 adults with CKD showed that the source of carbohydrate was strongly associated with mortality patterns. Per 100 g/day higher intake, carbohydrates from whole grains (HR = 0.81), raw fruits (HR = 0.60) and vegetables (HR = 0.66) were each linked to lower all-cause mortality risk. Parallel inverse associations were observed for cardiovascular mortality with raw fruits (HR = 0.71) and vegetables (HR = 0.58). Substitution analyses further indicated that replacing fat with carbohydrates from whole grains, raw fruits or vegetables (HRs 0.86, 0.79 and 0.82, respectively) lowered all-cause mortality, whereas substitutions for protein intake had no benefit [83]. Taken together, these observations describe cross-sectional associations between fibre-rich carbohydrate sources and mortality endpoints in CKD cohorts. However, given the inherent limitations of observational data, including differences in study design and the use of adjustments that may involve factors on the causal pathway, these findings should be interpreted as hypothesis-generating rather than conclusive. The overall direction of association nevertheless aligns with the mechanistic and biochemical improvements observed in interventional studies, supporting cautious interpretation but reaffirming the need for adequately powered, long-duration trials with hard clinical endpoints.

### 4.1. Clinical Implications of the Findings

Across CKD stages, the findings of this review indicate several practical implications for renal nutrition care. Where possible, dietary fibre should be obtained primarily from foods. Clinical implications of this review indicate that fibre use in CKD should follow a modality-specific, dose- and duration-dependent approach. In predialysis CKD, inulin 10 g/day for 12 weeks and β-glucan ~3 g/day for 14 weeks are the most practical options for reducing inflammatory tone and free toxin fractions, whereas RS2 15–33 g/day for up to 16 weeks is useful primarily when selective pCS reduction is desired. In haemodialysis, RS2 at 16–25 g/day for 8–16 weeks is the most widely supported intervention for attenuating cytokine-mediated inflammation and improving pCS when longer courses are used, while inulin or β-glucan for 10–14 weeks may be considered when toxin management is prioritised. In peritoneal dialysis, short-term supplements are largely biochemically neutral. However, ITF 10 g/day for 12 weeks and FOS 20 g/day for 74 days may be used to support fermentative balance or manage constipation. Across modalities, dietary fibre intake ≥20–25 g/day should be encouraged where potassium tolerance allows, as part of a personalised renal nutrition strategy. All interventions require a gradual increase and monitoring of potassium, gastrointestinal tolerance and overall energy and protein adequacy. In addition, isolated findings such as the rise in parathyroid hormone observed in the β-feasibility study highlight the importance of routine monitoring of calcium–phosphate balance during higher-dose or prolonged fibre supplementation, particularly in predialysis CKD [52]. The characteristics of the dietary fibre interventions evaluated across CKD stages, including fibre type, dose and duration, are summarised in Table 7.

### 4.2. Limitations of the Study

This systematic review has several limitations. First, although the search strategy was comprehensive, the exclusion of non-English publications may have introduced language bias and resulted in the omission of relevant international evidence. Second, heterogeneity in study design, fibre types (soluble vs. insoluble), dosage, and outcome measures may affect the comparability across trials and limit the generalisability of findings. Third, while the majority of toxin-related and inflammatory outcomes were derived from randomised controlled trials, several domains—particularly habitual diet and long-term kidney function—relied on observational evidence, which restricts causal inference. Fourth, the lack of standardised reporting across studies, particularly regarding dietary assessment methods and CKD staging, may introduce variability in interpretation. Possible publication bias cannot be excluded, as most included trials were small, short-term and tended to report positive biomarker changes, with few null-effect studies available. Selective outcome reporting may also be present, as several studies provided only partial biomarker data or incomplete toxin-fraction reporting. Moreover, access to some full-text publications from our institution was limited, which may have reduced the completeness of the evidence base and contributed to potential selection bias.

Additionally, interpretation is constrained by the absence of explicit recommendations on dietary fibre type, dosage and duration in major renal nutrition guidelines, including KDIGO 2024 [84] and KDOQI 2020 [85], limiting the ability to benchmark findings against established clinical standards. Dietary potassium restrictions in advanced CKD may also complicate the practical adoption of fibre-rich diets.

From a clinical nutrition perspective, increasing dietary fibre intake in patients with CKD presents several challenges. Although fibre offers multiple metabolic and gastrointestinal benefits, its incorporation into the diet must be undertaken gradually and with close clinical monitoring. A sudden increase in fibre consumption may precipitate gastrointestinal symptoms such as bloating, excessive gas, abdominal discomfort, or diarrhoea, all of which can negatively affect patient adherence. Moreover, many fibre-rich foods, including fruits, vegetables, legumes, and whole grains, are naturally rich in potassium. For individuals with impaired kidney function, excessive potassium intake may increase the risk of hyperkalaemia. Consequently, specific food-preparation strategies, such as soaking, sprouting, fermenting, discarding cooking water, and double-boiling techniques, may be necessary to reduce the potassium content of fibre-rich foods.

Patients with reduced appetite or advanced CKD may also experience early satiety when consuming high-fibre meals, making it more difficult to achieve adequate energy and protein intake. This, in turn, may exacerbate the risk of protein-energy wasting (PEW). Additionally, fibre can interfere with the absorption of certain nutrients and medications, necessitating careful timing of fibre intake relative to prescribed treatments. For these reasons, dietary fibre should be introduced in a personalised, stepwise manner, tailored to the patient’s clinical condition, dietary restrictions, and individual tolerance. Close collaboration between nephrologists and renal dietitians is essential to ensure that fibre intake is optimised safely and effectively within the broader context of CKD nutritional management.

This review was registered in PROSPERO, reducing the risk of selective reporting.

## 5. Conclusions

Dietary fibre may support improvements in inflammation, reductions in uraemic toxins, and aspects of kidney function in individuals with CKD. Despite these potential benefits, fibre remains underrepresented in CKD dietary guidelines. Further clinical trials are required to confirm its therapeutic role and to inform personalised nutrition strategies. Based on the evidence synthesised in this review, future guidelines should consider setting minimum fibre intake recommendations tailored to CKD stages and potassium tolerance. The use of composite dietary metrics, such as the DI-GM index and the P/F ratio, may support individualised dietary planning. Additionally, potassium restrictions should be carefully managed to avoid unintended reductions in fibre intake. Promoting plant-based, fibre-rich diets and educating patients on safe sources of dietary fibre may support metabolic health in CKD, although evidence for effects on long-term kidney outcomes remains limited. This review links practical health outcomes with biological explanations, helping to address an important gap in CKD nutrition research and encouraging the use of fibre-based approaches in personalised dietary care.

More high-quality, long-duration clinical trials are needed to clarify the benefits of dietary fibre in CKD management and to explore its impact on a broader range of uraemic toxins [11]. Increasing dietary fibre intake in CKD patients offers multiple favourable metabolic and gastrointestinal effects, including improved gut microbiota, reduced inflammation, and lower uraemic toxin levels. Fibre may represent a supportive component of CKD dietary management, with selective benefits observed for inflammatory markers and uraemic toxins, while kidney function endpoints were generally unchanged in short-term trials.

## Figures and Tables

**Table 1:** Dietary Inflammatory Index (DII) scores and corresponding dietary patterns.

DII Score	Meaning	Diet Type
<0	anti-inflammatory diet	high in fibre, fruits, vegetables, omega-3s
=0	neutral	balanced intake
>0	pro-inflammatory diet	high in saturated fats, sugar, processed foods

**Table 2:** Effects of dietary fibre on inflammatory outcomes.

Reference	Study Design	Exposure	Main Findings
Azevedo (2020) [28] RoB: high risk	RCT DB, HD (*n* = 31)	HAM-RS2 16 g/day, 4 weeks	↓NF-kB: 1.35–0.97 mg/L (−28%) (NS) ↔IAA
Esgalhado (2018) [29] RoB: some concerns	RCT DB, HD (*n* = 20)	HAM-RS2 16 g/day, 4 weeks	↓IL-6: 113.0→99.6 pg/mL (−12%) ↔hs-CRP ↓TBARS: 4.4→2.0 mmol/mL (−55%)
de Paiva (2020) [30] RoB: some concerns	RCT DB, HD (*n* = 16)	HAM-RS2 16 g/day, 4 weeks	↓RANTES: 545.0→312.2 pg/mL (−43%) ↓IP-10: 1121.2→556.2 pg/mL (−50%) ↓PDGF-BB: 326.9→137.2 pg/mL (−58%)
Laffin (2019) [31] RoB: some concerns	RCT DB, HD (*n* = 20)	HAM-RS2 20 g/day, 4 weeks 25 g/day, 4 weeks	↓IL-6: 157.52→118.52 ng/mL (−25%) ↓TNF-α: 318.69→261.26 ng/mL (−18%)
Khosroshahi (2018) [32] RoB: low	RCT DB, HD (*n* = 44)	HAM-RS2 20 g/day, 4 weeks 25 g/day, 4 weeks	↓IL-6: 150→100 ng/mL (−33%) ↓TNF-α: 250→220 ng/mL (−12%) ↓MDA: 1.6→1.4 µmol/L (−12%) ↔hs-CRP
Khosroshahi (2019) [33] RoB: low	RCT DB, HD (*n* = 44)	HAM-RS2 20 g/day, 4 weeks 25 g/day, 4 weeks	↔hs-CRP no inflammatory effect in either group
Xie (2015) [13] RoB: some concerns	RCT, HD (*n* = 124)	group A (10 g/day) group B (20 g/day) 75% digestible water-soluble fibre 6 weeks	Group A ↓CRP: 10.7→4.8 mg/L (−55%) Group B ↓CRP:9.8→4.7 mg/L(−52%) Group A ↓IL-6: 47.2→31.8 pg/mL(−33%), Group B ↓IL-6: 48.7→35.2 pg/mL, (−28%) Group A ↓IL-8: 94.8→22.2 pg/mL(−77%) Group B ↓IL-8: 91.4→34.5 pg/mL(−62%) Group A ↓TNF-α: 13.3→10.1 pg/mL (−24%) Group B ↓TNF-α: 12.5→10.6 pg/mL (−15%) Group A ↓MDA: 15.4→6.4 mmol/L (−58%) Group B ↓MDA: 12.5→5.6 mmol/L (−55%) Group A ↑T-AOC (GA: from 7.3→16.4 U/mL (+125%) Group B ↑ (GB: from 6.4→15.7 U/mL, ↑~145%)
Cui (2024) [34] RoB: low	RCT DB, PD (*n* = 40)	15 g/day SDF mix (inulin, resistant dextrin, FOS, GOS, XOS) 30 days	↓IL-8 in SDF ↑IL-18 in placebo ↔IL-6, TNF- α, IFN-ɣ, IL-1β
Pajk (2025) [35] RoB: some concerns	RCT, PD (*n* = 21)	dietary counselling on Mediterranean diet 4 weeks	↔IL-6 ↔TMAO
Gao (2020) [36] RoB: serious	non-randomised crossover trial, PD (*n* = 8)	16 g/day p-inulin 8 weeks (24-weeks trial)	↔hs-CRP ↔IL-6 ↔TNF-α ↓sCD14: 4267→4110 mg/mL (−4%)
de Andrade (2021) [37] RoB: some concerns	crossover RCT, PD (*n* = 26)	unripe banana flour (48% RS) 5 g RS 3 days 10 g RS ~4 weeks	↔hs-CRP ↔IL-6 ↔TNF-α ↔IL-10 ↔LPS dose/adherence limited
Headley (2025) [38] RoB: some concerns	RCT DB, CKD 3a–4 (*n* = 55)	HAM-RS2 15 g/day, 1 week 33 g/day, 15 weeks	↔hs-CRP, IL-6, TNF-α, IL-10 ↔MDA no inflammatory change reported
Chang (2023) [39] RoB: some concerns	RCT DB, CKD 4–5 (*n* = 54→45)	diet + inulin 10 g/day, 12 weeks	↔hs-CRP ↓IL-6: 23.92→10.94 pg/mL (−54%) ↔TNF-α
Sohn (2024) [40] RoB: serious	non-randomised CKD 3–4 trial (*n* = 15→13)	16 g/day p-inulin 12 weeks (28-week trial)	Week 8-week 20 ↔CRP ↔IL-6 ↔TNF-α
Elamin (2017) [41] RoB: high	RCT, CKD pre–post (*n* = 36→30)	10/20/40 g/day Gum Arabic 4 weeks	drop in CRP for each dose group ↓CRP: 3.5→2.8 ng/mL (−20%) ↔IL-6 ↔TNF-α ↔other inflammatory markers
Kwon (2024) [42] RoB: some concerns	crossover RCT, CKD 3–4 (*n* = 50→46)	Korean Mediterranean high-fibre diet (Medi-POB)	↓GM-CSF (NS) ↔IFN-ɣ, IL-1β, IL2, IL-4, IL-5, IL-6, IL-8, IL-10, TNF-α, IL-12p70, VEGF no evidence that MeDi-POB diet alters GM-CSF decreased significantly within the MEDi-POB period, with no significant between-diet difference
Pan (2025) [12] RoB: moderate	retrospective comparative, CKD (*n* = 145)	higher fibre intake (~26 g/day vs. 24 g/day)	low DII group vs. high DII group ↓CRP 3.85 vs. 4.59 mg/L ↓TNF-α: 8.91 vs. 3.55 pg/mL ↓fibrinogen: 3.55 vs. 3.89 g/L
Lai (2019) [43] RoB: serious	prospective controlled interventional study CKD 3–4 (*n* = 16)	LPD +19 g/day inulin 6 months	↓CRP (*p* = 0.003) ↔IL-6 ↓TNF-α: 171.2–116.2 µg/L ↔IL-1β ↓NOX2: 0.67–0.58
Lu (2017) [44] RoB: low to moderate	Cohort, CKD 3–4 (*n* = 157)	<25 g/d low fibre >25 g/d high fibre <61 g/d low protein >61 g/d high protein 18-month follow-up	high fibre ↑IL-6: 1.33→1.54 pg/mL (+16%) low fibre ↑IL-6: 1.55→1.95 pg/mL (+26%) LPLF ↑IL-6: 1.56→2.06 pg/mL (+32%) LPHF ↑IL-6: 1.42→1.78 pg/mL (+25%) HPLF ↑IL-6: 1.78→2.07 pg/mL (+16%) HPHF ↑IL-6: 1.38→1.63 pg/mL (+18%) high fibre ↑CRP: 2.63→2.94 mg/mL (+12%) low fibre ↑CRP: 3.43→4.16 mg/mL (+21%) LPLF ↑CRP: 4.08→4.69 mg/mL (+15%) LPHF ↑CRP: 2.97→3.52 mg/mL (+18%) HPLF ↑CRP: 3.67→4.21 mg/mL (+15%) HPHF ↑CRP: 2.71→3.28 mg/mL (+21%)
Kaesler (2021) [23] RoB: moderate	cross-sectional CKD 3–4 GCKD cohort (*n* = 3193)	continuous dietary fibre	↑fibre → ↓CRP (β = −0.10, *p* < 0.001)
Krishnamurthy (2012) [45] RoB: moderate	cross-sectional observational analysis (*n* = 14 543 all 1 105 with CKD 3–4)	total fibre soluble fibre insoluble fibre per 10 g/day	for each 10 g/day: total higher fibre intake CKD subgroup: 38% lower odds (OR = 0.62) higher insoluble fibre intake CKD subgroup: 45% lower odds (OR = 0.55) higher soluble fibre intake CKD subgroup: 74% lower odds (OR = 0.26) fibre intake inversely associated with inflammation, especially in CKD
Udomkarnjananun (2025) [46] RoB: serious	cross-sectional CKD 3–4 (*n* = 135)	dietary patterns: LPLF, LPHF, HPLF, HPHF >0.8 g/kg high protein <0.8 g/kg high protein median 8 g/day fibre	LPHF vs. HPLF ↓IL-18: 408.5 vs. 854 ng/mL ↓MCP-1: 288.8 vs. 416.2 ng/mL
Xu (2014) [24] RoB: low to moderate	cross-sectional +prospective, ULSAM cohort (*n* = 1100 men)	fibre quartiles Q1 ≤ 14.5 g/day Q2 14.5–16.8 g/day Q3 16.8–19.2 g/day Q4 >19.2 g/day	35% ↓odds of having elevated CRP (>3 mg/L) (Q4 OR = 0.65, 95% CI: 0.43–0.98, *p* = 0.04)
Xu (2015) [25] RoB: low to moderate	cross-sectional, ULSAM + PIVUS (*n* = 1942)	ADII	for each 1SD ↑ADII (3.26 units) → ↑CRP(6%)
Xu (2016) [26] RoB: low	prospective cohort ULSAM (*n* = 390 men)	P/F ratio	↑P/F → ↑CRP (rho = 0.13, *p* < 0.05), positive correlation with inflammation
Hosseini (2020) [27] RoB: low to moderate	cross-sectional; adults (*n* = 2125)	≥5 servings fruit/veg	↓hsCRP in smokers (OR = 0.10, 95% CI:0.04–0.21) 90% lower odds of elevated CRP

↑ increase, ↓ decrease, ↔ no effect, no significant change, unchanged, (NS)—non-significant, (n = 54→45) reduction in the number of study participants, PD—peritoneal dialysis, HD—haemodialysis, NDD—non-dialysis-dependent, NFkB—nuclear factor kappa-light-chain-enhancer of activated B cells, IL-6—interleukin 6, TBARS—thiobarbituric acid reactive substances, MDA—malondialdehyde, LPS—lipopolysaccharide, RANTES/CCL5—Regulated Upon Activation, Normal T-cell Expressed and Secreted/C-C Motif Chemokine Ligand 5, IP-10/CXCL10—Interferon-ɣ-inducible protein-10, PDGF-BB—platelet-derived growth factor, BB isoform.

**Table 3:** Effects of dietary fibre on uraemic toxins outcomes.

Reference	Study Design	Exposure	Main Findings
Azevedo (2020) [28] RoB: high risk	RCT DB, HD (*n* = 31)	HAM-RS2 16 g/day, 4 weeks	↔IAA: 2132→1917 mg/L (−11%), (NS) ↔AhR expression
Esgalhado (2018) [29] RoB: some concerns	RCT DB, HD (*n* = 20)	HAM-RS2 16 g/day, 4 weeks	↓IS: 27→22.1 mg/L (−18%) ↔pCS
Khosroshahi (2019) [33] RoB: low	RCT DB, HD (*n* = 44)	HAM-RS2 20 g/day, 4 weeks 25 g/day, 4 weeks	↔IS ↓p-cresol: 7.8→5.34 µmol/L (−31%)
Ebersolt (2022) [49] RoB: moderate	observational, HD (*n* = 58)	fibre intake, P/F index	↑fibre→↓IS (r = −0.56, *p* = 0.015) ↑fibre→↓pCS (r = −0.47, *p* = 0.041) (pCS in anuric patients only) ↑P/F ratio & ↑toxins ↑P/F ratio→↑IS (r = 0.47, *p* = 0.048) ↑P/F ratio→↑pCS (r = 0.46, *p* = 0.052)
Cui (2024) [34] RoB: low	RCT DB, PD (*n* = 40)	15 g/day SDF mix (inulin, resistant dextrin, FOS, GOS, XOS) 30 days	↔TMAO, toxin effects absent within or between groups
de Andrade (2021) [37] RoB: some concerns	crossover RCT, PD (*n* = 26)	unripe banana flour (48% RS) 5 g RS 3 days 10 g RS ~4 weeks	↔IS, ↔pCS, ↔IAA (dose limited adherence)
Li (2020) [50] RoB: some concerns	crossover RCT, PD (*n* = 15)	10 g/day ITF 12 weeks (inulin-type fructans: 50% long chain inulin+ 50% oligofructose)	ITF restricted the ↑ in faecal indole ↔faecal p-cresol ↔serum IS ↔serum pCS: 8.75–3.38 mg/L (−61%), (NS) ↔urinary&dialisate toxins
Gao (2020) [36] RoB: serious	non-randomised crossover trial, PD (*n* = 8)	16 g/day p-inulin 8 weeks (24-week trial)	week 8–week16 IS (*p* < 0.0001) pCS (*p* = 0.004) TMAO (*p* < 0.0001)
Headley (2025) [38] RoB: some concerns	RCT DB, CKD 3a–4 (*n* = 55)	HAM-RS2 15 g/day, 1 week 33 g/day, 15 weeks	↔IS ↓pCS (~23%)
Chang (2023) [39] RoB: some concerns	RCT DB, CKD 4–5 (*n* = 54→45)	diet + inulin 10 g/day, 12 weeks	↓IS: 3.42→2.83 µg/mL (−17%) ↓pCS: 7.52→4.02 µg/mL (−46%)
Ebrahim (2022) [47] RoB: some concerns	RCT, CKD 3–5 (*n* = 45)	13.5 g supplement (22.5% (~3 g) β-glucan) 14 weeks	↓free IS: 0.35, 95% CI: 0.21–0.60 (−65%) ↓free pCS: 0.48, 95% CI: 0.29–0.82 (−52%) ↓free pCG: 0.13, 95% CI: 0.06–0.27 (−87%) ↓total pCG: 0.14, 95% CI: 0.08–0.28 (−86%) also trend (NS) at week 14 only free IAA ↓free IAA: 0.56, 95% CI: 0.31–1.00 (−44%)
Ramos (2019) [51] RoB: low	RCT DB, CKD 3–5 (*n* = 46)	FOS 3 g, 3 days 6 g, 3 days 9 g, 3 days 12 g, until 3 months	trend towards reduction in serum total pCS ↔total pCS: (−11%), (NS)
Poesen (2016) [48] RoB: low	crossover RCT, CKD 3–4 (*n* = 40)	10 g/day AXOS 4 weeks	↔IS ↔pCS ↔pCG, ↓TMAO: 0.789, 95% CI: 0.629–0.990 (−21%)
Sohn (2024) [40] RoB: serious	non-randomised CKD 3–4 trial (*n* = 15→13)	16 g/day p-inulin 12 weeks (28-week trial)	↔IS ↔pCS
Hill (2020) [52] RoB: serious	non-randomised pre–post, CKD 3–4 (*n* = 18)	3 g/day β-glucan 12 weeks	↔IS ↔pCS ↓TMAO: 14.9–10.7 µmol/L (−28%)
Salmean (2015) [53] RoB: serious	non-randomised intervention CKD 3a–5 (*n* = 13)	10 g/day pea hull fibre 4 weeks (10 g PHF + 15 g inulin)/day for 4 weeks	↓p-cresol: 7.25–5.82 mg/L (−20%)
Lu (2017) [44] RoB: low to moderate	cohort, CKD 3–4 (*n* = 157)	<25 g/day low fibre >25 g/day high fibre <61 g/da low protein >61 g/day high protein 18-month follow-up	high fibre ↑IS: 23.6→31.1 ng/mL (+32%) low fibre ↑IS: 30.9→40.1 ng/mL (+30%) LPLF ↑IS: 27.9→37.4 ng/mL (+34%) LPHF ↑IS: 26.2→35.1 ng/mL (+34%) HPLF ↑IS: 35.5→50.6 ng/mL (+42%) HPHF ↑IS: 29.0→33.7 ng/mL (+16%)
Rossi (2015) [54] RoB: low to moderate	cross-sectional CKD 4–5 (*n* = 40)	fibre intake, P/F index	↑total fibre→↓free pCS (r = −0.42, *p* = 0.007) ↑total fibre→↓total pCS (r = −0.44, *p* = 0.04) ↑soluble fibre→↓total pCS (r = −0.42, *p* = 0.007) ↑insoluble fibre→↓total pCS (r = −0.42, *p* = 0.007) ↑P/F→↑free IS (r = 0.34, *p* = 0.031) ↑P/F→↑free pCS (r = 0.40, *p* = 0.012) ↑P/F→↑total pCS (r = 0.43, *p* = 0.005)
Ramos (2020) [55] RoB: moderate	cross-sectional NDD CKD (*n* = 43)	constipation phenotype BSS classification hard stools BSS < 3 normal stools: BSS ≥ 3 ROME III criteria constipated non-constipated	↔IS constipated (BSS < 3) vs. normal (BSS ≥ 3) total pCS: 318.4 vs. 195.2 µmol/L (+63%) free pCS: 3.7 vs. 2.0 µmol/L (+85%) urinary pCS: 1061.3 vs. 659.1 µmol/L (+61%) ROME III criteria (trend only): total pCS: 318.4 vs. 199.6 µmol/L (+59%) (NS) ↔free pCS urinary pCS: 1061.3 vs. 724.2 µmol/L (+46%) (NS) ↑BSS < 3 → ↑total pCS (r = 1.54, *p* = 0.02) ↑BSS < 3 → ↑free pCS (r = 1.40, *p* = 0.05) ↑BSS < 3 → ↑urinary pCS (r = 1.77, *p* = 0.02)
Udomkarnjananun (2025) [46] RoB: serious	cross-sectional CKD 3–4 (*n* = 135)	dietary patterns: LPLF, LPHF, HPLF, HPHF >0.8 g/kg high protein <0.8 g/kg high protein median 8 g/day fibre	↔IS ↔pCS ↓TMAO: LPHF vs. HPLF
El Amouri (2021) [56] RoB: low to moderate	longitudinal mixed model paediatric CKD 1–5 (*n* = 61)	low fibre < 11.7 low fibre ≥ 11.7 [g/day/m^2^] fibre increase (per 1 g/day)	low-fibre group vs. high-fibre group ↔IS ↔pCS ↓total pCG: 0.009 vs. 0.004 mg/dL ↓free pCG: 0.008 vs. 0.003 mg/dL ↔IAA
El Amouri (2021) [57] RoB: low to moderate	cross-sectional paediatric CKD 1–5 (*n* = 61)	fibre (g/day/m^2^)	↔total IS ↓free IS: 0.969, 95% CI: 0.941–0.997 (−3%) ↔total pCS ↓free pCS: 0.975, 95% CI: 0.953–0.998 (−2%) ↓total IAA: 0.984, 95% CI: 0.971–0.997 (−2%) ↓free IAA: 0.934, 95% CI: 0.907–0.963 (−7%) ↓total pCG: 0.970, 95% CI: 0.944–0.99 (−3%) ↓free pCG: 0.967, 95% CI: 0.943–0.992 (−3%)

↑ increase, ↓ decrease, ↔ no effect, no significant change, unchanged, (NS)—non-significant, PD—peritoneal dialysis, HD—haemodialysis, NDD—non-dialysis-dependent, IS—indoxyl sulphate, pCS—p-cresyl sulphate, AhR—aryl hydrocarbon receptor, IAA—indole-3-acetic acid, LPS—lipopolysaccharide, TMAO—trimethylamine-N-oxide.

**Table 4:** Effects of dietary fibre on nutritional status outcomes.

Reference	Study Design	Exposure	Main Findings
Khosroshahi (2019) [33] RoB: low	RCT DB, HD (*n* = 44)	HAM-RS2 20 g/day, 4 weeks 25 g/day, 4 weeks	↔albumin ↔lipids ↔HGB ↔BMI fibre well tolerated
Esgalhado (2018) [29] RoB: some concerns	RCT DB, HD (*n* = 20)	HAM-RS2 16 g/day, 4 weeks	↔weight ↔BMI ↔fat mass ↔albumin ↔HGB
Jarupala (2023) [58] RoB: some concerns	RCT, HD (*n* = 250)	fibre-increasing dietary education	↑albumin ↑HGB ↑total protein
Li (2022) [59] RoB: high risk	RCT, HD (*n* = 162)	10 g/day SDF 8 weeks	↑HGB ↑Fe^2+^, ↑ferritin ↑SCFAs (butyrate, hexanoate)
Farman (2020) [60] RoB: critical!	pre–post, HD (*n* = 50)	30 g/day Gum Arabic 6 months	↑albumin ↑total protein ↓lipids
de Andrade (2021) [37] RoB: some concerns	crossover RCT, PD (*n* = 26)	unripe banana flour (48% RS) 5 g RS 3 days 10 g RS ~4 weeks	↔energy intake ↔protein intake ↔albumin ↔nPNA
Cui (2024) [34] RoB: low	RCT DB, PD (*n* = 40)	15 g/day SDF mix (inulin, resistant dextrin, FOS, GOS, XOS) 30 days	↔albumin ↔prealbumin ↔BMI ↔energy intake high dietary adequacy issues persisted
Meksawan (2016) [61] RoB: some concerns	crossover RCT, PD (*n* = 13→9)	20 g/day FOS 74 days	↔albumin ↔weight ↔lipids ↓constipation
Chang (2023) [39] RoB: some concerns	RCT DB, CKD 4–5 (*n* = 54→45)	diet + inulin 10 g/day, 12 weeks	↔albumin ↔prealbumin ↔transferrin no adverse nutritional effects
Tayebi-Khosroshahi (2016) [62] RoB: some concerns	RCT, CKD 3–4 (*n* = 32)	90 mL lactulose/day	↔HGB ↔albumin GI tolerance acceptable
Poesen (2016) [48] RoB: low	crossover RCT CKD 3–4 (*n* = 40)	10 g/day AXOS 4 weeks	↔body weight ↔glucose, HOMA-IR ↔lipids
Khalid (2021) [63] RoB: serious	single-arm, CKD 2–3 (*n* = 68→59)	25 g/day Gum Arabic 12 months	↔HGB no nutritional harm
Salmean (2015) [53] RoB: serious	non-randomised intervention CKD 3a–5 (*n* = 13)	10 g/day pea hull fibre 4 weeks (10 g PHF + 15 g inulin)/day for 4 weeks	↔SNAQ ↔apetite ↔weight ↔serum markers ↓cholesterol
Hill (2020) [52] RoB: serious	non-randomised pre–post, CKD 3–4 (*n* = 18)	3 g/day β-glucan 12 weeks	↔weight ↔BMI ↔albumin ↔nutritional markers
Lu (2017) [44] RoB: low to moderate	cohort, CKD 3–4 (*n* = 157)	<25 g/day low fibre >25 g/day high fibre <61 g/da low protein >61 g/day high protein 18-month follow-up	↔albumin ↔prealbumin ↔BMI ↔MAMC

↑ increase, ↓ decrease, ↔ no effect, no significant change, unchanged, (NS)—non-significant, PD—peritoneal dialysis, HD—haemodialysis, NDD—non-dialysis dependent.

**Table 5:** Effects of dietary fibre on kidney function outcomes.

Reference	Study Design	Exposure	Main Findings
Khosroshahi (2019) [33] RoB: low	RCT DB, HD (*n* = 44)	HAM-RS2 20 g/day, 4 weeks 25 g/day, 4 weeks	↓creatinine ↓uric acid ↓urea (NS)
Khosroshahi (2018) [32] RoB: low	RCT DB, HD (*n* = 44)	HAM-RS2 20 g/day, 4 weeks 25 g/day, 4 weeks	↓BUN ↓creatinine ↓eGFR(indirectly)
Esgalhado (2018) [29] RoB: some concerns	RCT DB, HD (n = 20)	HAM-RS2 16 g/day, 4 weeks	↔creatinine ↔urea ↔albumin
Azevedo (2020) [28] RoB: high risk	RCT DB, HD (*n* = 31)	HAM-RS2 16 g/day, 4 weeks	↔creatinine ↔urea
Farman (2020) [60] RoB: critical!	pre–post, HD (*n* = 50)	30 g/day Gum Arabic 6 months	↓urea ↓creatinine ↓uric acid
Kemp (2021) [68] RoB: high risk	RCT DB, HD (*n* = 20)	HAM-RS2 16 g/day, 4 weeks	↔creatinine ↔urea
Headley (2025) [38] RoB: some concerns	RCT DB, CKD 3a-4 (*n* = 55)	HAM-RS2 15 g/day, 1 week 33 g/day, 15 weeks	↔eGFR ↔creatinine ↔BUN long duration did not alter renal marker
Chang (2023) [39] RoB: some concerns	RCT DB, CKD 4–5 (*n* = 54→45)	diet + inulin 10 g/day, 12 weeks	↔eGFR ↔serum creatinine ↔urea
Poesen (2016) [48] RoB: low	crossover RCT CKD 3–4 (*n* = 40)	10 g/day AXOS 4 weeks	↔eGFR ↔creatinine ↔urea
Kwon (2024) [42] RoB: some concerns	crossover RCT CKD 3–4 (*n* = 50→46)	Korean Mediterranean high-fibre diet (Medi-POB)	eGFR preserved vs. decline on control diet
Khalid (2021) [63] RoB: serious	single-arm, CKD 2–3 (*n* = 68→59)	25 g/day Gum Arabic 12 months	eGFR decline reversed during intervention (+improvement in ΔeGFR vs. baseline)
Lai (2019) [43] RoB: serious	prospective controlled interventional study CKD 3–4 (*n* = 16)	LPD +19 g/day inulin 6 months	↔eGFR ↓uric acid (*p* = 0.018) ↑bicarbonate in both groups
Lu (2017) [44] RoB: low to moderate	cohort, CKD 3–4 (*n* = 157)	<25 g/day low fibre >25 g/day high fibre <61 g/da low protein >61 g/day high protein 18-month follow-up	high fibre ↑GFR: 51.7→39.5 mL/min (−24%) low fibre ↑GFR: 50.6→36.1 mL/min (−29%) LPLF ↑GFR: 48.4→33.0 mL/min (−32%) LPHF ↑GFR: 52.1→39.6 mL/min (−24%) HPLF ↑GFR: 51.5→37.3 mL/min (−28%) HPHF ↑GFR: 51.6→38.0 mL/min (−26%) slower eGFR decline over time
Udomkarnjananun (2025) [46] RoB: serious	cross-sectional CKD 3–4 (*n* = 135)	dietary patterns: LPLF, LPHF, HPLF, HPHF >0.8 g/kg high protein <0.8 g/kg high protein median 8 g/day fibre	↔eGFR (LP-HF vs. HP-LF) ↔creatinine
Ramos (2020) [55] RoB: moderate	cross-sectional NDD CKD (*n* = 43)	constipation phenotype BSS classification hard stools BSS < 3 normal stools BSS ≥ 3 ROME III criteria constipated non-constipated	for constipated (BSS < 3) ↑eGFR → ↑total pCS (β = 0.95, *p* < 0.01) ↑eGFR → ↑free pCS (β = 0.93, *p* < 0.01)
Cui (2024) [34] RoB: low	RCT DB, PD (*n* = 40)	15 g/day SDF mix (inulin, resistant dextrin, FOS, GOS, XOS) 30 days	↔creatinine ↔cystatin C ↔urea ↔eGFR short duration
Li (2020) [50] RoB: some concerns	crossover RCT, PD (*n* = 15)	10 g/day ITF 12 weeks (inulin-type fructans): 50% long chain inuli+ 50% oligofructose)	↔residual GFR ↔creatinine clearance ↔Kt/V ↔ultrafiltration
Meksawan (2016) [61] RoB: some concerns	crossover RCT, PD (*n* = 13→9)	20 g/day FOS 74 days	↓eGFR ↓residual renal function small ↓urea nitrogen (NS)

↑ increase, ↓ decrease, ↔ no effect, no significant change, unchanged, (NS)—non-significant, PD—peritoneal dialysis, HD—haemodialysis, NDD—non-dialysis-dependent.

**Table 6:** Effects of dietary fibre on gut–liver–kidney axis outcomes.

Reference	Study Design	Exposure	Main Findings
Laffin (2019) [31] RoB: some concerns	RCT DB, HD (*n* = 20)	HAM-RS2 20 g/day, 4 weeks 25 g/day, 4 weeks	↑*Faecalibacterium* (0.40–3.21%) trend↑ *Prevotella*, ↔other genera
Khosroshahi (2018) [32] RoB: low	RCT DB, HD (*n* = 44)	HAM-RS2 20 g/day, 4 weeks 25 g/day, 4 weeks	constipation improved (↑bowel movement frequency)
Li (2022) [59] RoB: high risk	RCT, HD (*n* = 162)	10 g/day SDF (galactomannan, resistant dextrin, fructooligosaccharide and starch) 8 weeks	↑SCFAs (butyrate&hexanoate) ↑*Lactobacillus*, *Lactobacillaceae* and *Bifidobacterium* adolescentis
Esgalhado (2018) [29] RoB: some concerns	RCT DB, HD (*n* = 20)	HAM-RS2 16 g/day, 4 weeks	↔microbiota composition
Jarupala (2023) [58] RoB: some concerns	RCT, HD (n = 250)	fibre-increasing dietary education	improved diet quality improved fibre intake
Kemp (2021) [68] RoB: high risk	RCT DB, HD (*n* = 20)	HAM-RS2 16 g/day, 4 weeks	regulated taxa: *Oscillosperaceae*, *Roseburia*, *Ruminococcus gauvreauii*, downregulated: *Ruminococcus Chapanellens*, *Dialister*, *Coprococcus*, among others
Ebrahim (2022) [47] RoB: some concerns	RCT, CKD 3–5 (*n* = 45)	13.5 g supplement (22.5% (~3 g) β-glucan) 14 weeks	shift in overall microbiome composition (redundancy analysis *p* = 0.002) greater temporal stability trend ↑ *Prevotella*
Poesen (2016) [48] RoB: low	crossover RCT CKD 3–4 (*n* = 40)	10 g/day AXOS 4 weeks	↑flatluence ↔stool frequency FOS-like fermentability
Hill (2020) [52] RoB: serious	non-randomised pre–post, CKD 3–4 (*n* = 18)	3 g/day β-glucan 12 weeks	↔SCFAs, ↔gut taxa not reported, suggests microbiota-mediated toxin modulation
Tayebi-Khosroshahi (2016) [62] RoB: some concerns	RCT, CKD 3–4) (*n* = 32)	90 mL lactulose/day	↑*Bifidobacteria*, *Lactobacilli*
Sohn (2024) [40] RoB: serious	non-randomised CKD 3–4 trial (*n* = 15→13)	16 g/day p-inulin 12 weeks (28-week trial)	α- and β-diversity changed 30 genera altered across phases: ↑*Bifidobacterium* ↑*Anaerostipes* ↓*Lachnispira* ↓*Moryella* ↓*Negativibacillus* ↓*Ruminococcaceae* ↓*Erysipelotrichaceae*
Lai (2019) [43] RoB: serious	prospective controlled interventional study CKD 3–4 (*n* = 16)	LPD +19 g/day inulin 6 months	↑*Bifidobacteriaceae* ↓*Enterobacteriaceae*
Salmean (2013) [69] RoB: serious	single-blind rolling admission study CKD 3–5 (*n* = 17→15)	<2 g/day fibre 2 weeks 23 g/day pea hull fibre 4 weeks	a statistically significant increase in stool frequency: 1.3→1.6 stools per day, *p* = 0.02 total cholesterol improvement ↓TC: 175 to 167 mg/dL
Udomkarnjananun (2025) [46] RoB: serious	cross-sectional CKD 3–4 (*n* = 135)	dietary patterns: LPLF, LPHF, HPLF, HPHF >0.8 g/kg high protein <0.8 g/kg high protein median 8 g/day fibre	CKD vs. controls α-diversity unchanged β-diversity shifts vs. controls LPHF vs. HPLF ↑SCFA producers (*Lachnospiraceae NK4A136*, *Eubacterium ruminantium*) ↓proteolytic genera (*Klebsiella*, *Clostridium sensu stricto 1*)
Li (2020) [50] RoB: some concerns	crossover RCT, PD (*n* = 15)	10 g/day ITF 12 weeks (inulin-type fructans: 50% long-chain inulin+ 50% oligofructose)	↓*Bacteroides thetaiotaomicron* ↓faecal pH ITF restricted ↑indole levels ↔p-cresol bacteria
Cui (2024) [34] RoB: low	RCT DB, PD (*n* = 40)	15 g/day SDF mix (inulin, resistant dextrin, FOS, GOS, XOS) 30 days	↔overall diversity, Changes in composition: ↑*Prevotellaceae*, *Klebsiella*, *Veillonella*, *Paraprevotella*, *Lachnospiraceae*, within SDF group: ↑*Clostridia*, *Oscillospirales*, *Cetobacterium* ↑propionate
de Andrade (2021) [37] RoB: some concerns	crossover RCT, PD (*n* = 26)	unripe banana flour (48% RS) 5 g RS 3 days 10 g RS ~4 weeks	↔intestinal permeability markers (LPS) ↔gut function
Meksawan (2016) [61] RoB: some concerns	crossover RCT, PD (*n* = 13→9)	20 g/day FOS 74 days	↓colonic transit time ↑stool frequency improved stool form gut function improved
Gao (2020) [36] RoB: serious	non-randomised crossover trial, PD (*n* = 8)	16 g/day p-inulin 8 weeks (24-week trial)	5 most abundant phyla in PD patients: *Firmicutes, Bacteroidetes, Actinobacteria, Proteobacteria*, and *Verrocomicrobia*; no significant changes in α-diversity; 86 bacteria strains changed with p-inulin, but overall structure remains patient-specific Significant positive correlation among *Bacteroides*, dietary fibre, and carbohydrate consumption

↑ increase, ↓ decrease, ↔ no effect, no significant change, unchanged, (PD—peritoneal dialysis, HD—haemodialysis, NDD—non-dialysis-dependent.

**Table 7:** Clinical implications of the findings.

Predialysis CKD (Stages 3–4)			
**Fibre Type**	Dose Duration	Expected Effects	Best Use
inulin	10 g/day 12 weeks	↓IL-6 ↓free toxin fractions	early CKD with metabolic inflammation and rising toxin level
β-glucan	~3 g/day 14 weeks	↓free IS, pCS, pCG	metabolically active toxin burden, well-tolerated, safe in nutrient-restricted diet
RS	15–33 g/day 16 weeks	selective ↓pCS	when pCS is the main therapeutic target not recommended for short-term inflammation control in predialysis CKD
Gum Arabic	10–40 g/day 4 weeks	small ↓CRP	exploratory finding
dietary patterns	Mediterranean style fibre-rich approach ≥4–12 weeks	maintenance or slower decline of eGFR	for metabolic improvement in CVD and obesity, for longer kidney trajectory durations
**Peritoneal Dialysis (PD)**			
**Fibre Type**	**Dose** **Duration**	**Expected** **Effects**	**Best Use**
ITF	10 g/day 12 weeks	lower faecal pH stabilised indole production	dysbiosis modulation to more saccharolytic, no expected changes in serum IS or pCS at 4–12 weeks
SFM	15 g/day >30 days	small improvement in inflammatory tone (IL-8 dynamics)	supportive, not primary therapy
FOS	20 g/day 74 days	improved stool frequency and transit	PD-related constipation
**Haemodialysis (HD)**			
**Fibre Type**	**Dose** **Duration**	**Expected** **Effects**	**Best Use**
RS2	16–25 g/day 8–16 weeks	↓IL-6, ↓TNF-α with longer courses: ↓pCS	chronic low-grade inflammation, high pCS burden, constipation
inulin β-glucan	10–14 g/day 10–14 weeks	modest toxin reduction (free fractions)	patients prioritising toxin management

↓ decrease.

## Data Availability

Data are contained within the article.

## Supplementary Materials

The following supporting information can be downloaded at https://www.mdpi.com/article/10.3390/nu18091341/s1.
