# Supplementary material for: Dietary Fibre and Chronic Kidney Disease: A Systematic Review of Effects on Inflammation, Uraemic Toxins, Nutritional Status, Kidney Function, and Gut–Liver–Kidney Axis Mechanisms
Source: Nutrients. 2026 Apr 24;18(9):1341. doi: 10.3390/nu18091341 (PMC13165172; doi:10.3390/nu18091341)
Supplement: Supplementary file 1 [file nutrients-18-01341-s001.zip › PRISMA-S Checklist.pdf]

PRISMA-S Checklist

Section/topic	#	Checklist item	Location(s) Reported
**INFORMATION SOURCES AND METHODS**			
Database name	1	Name each individual database searched, stating the platform for each.	Methods section 2.1
Multi-database searching	2	If databases were searched simultaneously on a single platform, state the name of the platform, listing all of the databases searched.	NA
Study registries	3	List any study registries searched.	NA
Online resources and browsing	4	Describe any online or print source purposefully searched or browsed (e.g., tables of contents, print conference proceedings, web sites), and how this was done.	NA
Citation searching	5	Indicate whether cited references or citing references were examined, and describe any methods used for locating cited/citing references (e.g., browsing reference lists, using a citation index, setting up email alerts for references citing included studies).	reference list of included studies screened; no forward citation searching
Contacts	6	Indicate whether additional studies or data were sought by contacting authors, experts, manufacturers, or others.	no contact with authors or experts was made to obtain additional studies or data
Other methods	7	Describe any additional information sources or search methods used.	No additional information sources or search

			methods were used
**SEARCH STRATEGIES**			
Full search strategies	8	Include the search strategies for each database and information source, copied and pasted exactly as run.	at the end of this checklist
Limits and restrictions	9	Specify that no limits were used, or describe any limits or restrictions applied to a search (e.g., date or time period, language, study design) and provide justification for their use.	at the end of this checklist
Search filters	10	Indicate whether published search filters were used (as originally designed or modified), and if so, cite the filter(s) used.	No
Prior work	11	Indicate when search strategies from other literature reviews were adapted or reused for a substantive part or all of the search, citing the previous review(s).	No search strategies from previous reviews were adapted or reused
Updates	12	Report the methods used to update the search(es) (e.g., rerunning searches, email alerts).	no search updates were performed
Dates of searches	13	For each search strategy, provide the date when the last search occurred.	at the end of this checklist
**PEER REVIEW**			
Peer review	14	Describe any search peer review process.	No search peer review was conducted
**MANAGING RECORDS**			
Total Records	15	Document the total number of records identified from each database and other information sources.	Scopus: 1973 PubMed:1264 Medline:1230
Deduplication	16	Describe the processes and any software used to deduplicate records from multiple database searches and other information sources.	by Rayyan ai

			Methods section 2.2 and 2.6

PRISMA-S: An Extension to the PRISMA Statement for Reporting Literature Searches in Systematic Reviews
Rethlefsen ML, Kirtley S, Waffenschmidt S, Ayala AP, Moher D, Page MJ, Koffel JB, PRISMA-S Group.
Last updated February 27, 2020.

Data limits applied in all databases. In Scopus, filters applied: English and article type. PubMed and Medline Complete: date limits only.

PubMed and Scopus last search on 31 Dec 2025, Medline Complete: last search on 17 Jan 2026.

- PubMed: (("Chronic Kidney Disease"[MeSH Terms] OR "chronic kidney disease" OR CKD OR "renal insufficiency" OR "kidney failure") AND ("Dietary Fiber"[MeSH Terms] OR "dietary fiber" OR "dietary fibre" OR fiber or fibre OR prebiotic OR "resistant starch" OR inulin OR "beta-glucan" OR psyllium))
- Scopus: TITLE-ABS-KEY((“chronic kidney disease” OR CKD OR “renal insufficiency” OR “kidney failure”) AND (“dietary fiber” OR “dietary fibre” OR fiber OR fibre OR prebiotic OR “resistant starch” OR inulin OR “beta-glucan” OR psyllium)).
- Medline Complete: (CKD or "chronic kidney disease" or "renal insufficiency" or "kidney failure") AND (fiber or fibre or "dietary fibre" or "dietary fiber" or prebiotic or "beta-glucan" or inulin)
