# Supplementary material for: Dietary Fibre and Chronic Kidney Disease: A Systematic Review of Effects on Inflammation, Uraemic Toxins, Nutritional Status, Kidney Function, and Gut–Liver–Kidney Axis Mechanisms
Source: Nutrients. 2026 Apr 24;18(9):1341. doi: 10.3390/nu18091341 (PMC13165172; doi:10.3390/nu18091341)
Supplement: Supplementary file 1 [file nutrients-18-01341-s001.zip › prospero_registration_preview.pdf]

Dietary Fibre and Chronic Kidney Disease: A Systematic Review of Effects on Inflammation, Uraemic Toxins, Nutritional Status, Kidney Function, and Gut–Liver–Kidney Axis Mechanisms

Anna Gabriela Mojak, Monika Bronkowska

Citation
Anna Gabriela Mojak, Monika Bronkowska. Dietary Fibre and Chronic Kidney Disease: A Systematic Review of Effects on Inflammation, Uraemic Toxins, Nutritional Status, Kidney Function, and Gut–Liver–Kidney Axis Mechanisms. Not yet published.

PREVIEW

REVIEW TITLE AND BASIC DETAILS

Review title
Dietary Fibre and Chronic Kidney Disease: A Systematic Review of Effects on Inflammation, Uraemic Toxins, Nutritional Status, Kidney Function, and Gut–Liver–Kidney Axis Mechanisms

Condition or domain being studied
Chronic Kidney Disease

Rationale for the review
While previous studies have examined the effects of dietary fibre on inflammation and uraemic toxins in CKD, they often focused solely on randomised trials or lacked mechanistic insights. This review will address a broader clinical and biological context by integrating observational and interventional human studies, excluding animal models, and exploring the gut-liver-kidney axis as a key pathway. It will update earlier reviews with recent evidence and aims to inform clinical practice and guide future research.

Review objectives
To systematically synthesise evidence on the effects of dietary fibre intake on: inflammatory markers, gut-derived uraemic toxins, kidney function, nutritional status

Keywords
Chronic kidney disease; Dietary fibre; Soluble fibre; Inflammation; Uraemic toxins; Nutritional status; Kidney function; gut-liver-kidney axis; Microbiota; Dietary inflammatory index; Systematic review

Country
Poland

ELIGIBILITY CRITERIA

Population

Included
patients with chronic kidney disease at various stages (1-5)

Excluded
specific exclusion criteria for the population have not yet been defined and will be determined during the screening process

Intervention(s) or exposure(s)

Included
The exposure of interest is dietary fibre from any source (e.g. food, supplements) assessed in relation to CKD outcomes. Studies that quantify dietary fibre intake and examine its association with kidney function, inflammation, uraemic toxins, or nutritional status will be included.

Excluded

Studies that do not assess any form dietary fibre intake at all or do not examine its association with CKD-related outcomes.

Comparator(s) or control(s)

This review does not have any comparators

Study design

Both randomized and nonrandomized study types will be included.

Included
Studies will be included if they:

- are original observational (cross-sectional, cohort, case-control) or interventional (RCT, non-randomised trials)
- are conducted exclusively on human subjects
- are published in English
- assess dietary fibre intake in relation to CKD outcomes
- report outcomes related to kidney function, inflammation, uraemic toxins, or nutritional status
- provide quantitative data

Excluded
Studies will be excluded if they:

- are review articles
- are conducted on animals
- do not specifically address both CKD and dietary fibre

Context

Studies must report dietary fibre intake and relevant outcomes in human populations diagnosed with CKD at any stage.

SIMILAR REVIEWS

Check for similar records already in PROSPERO

PROSPERO identified a number of existing PROSPERO records that were similar to this one (last check made on 30 December 2025). These are shown below along with the reasons given by that the review team for the reviews being different and/or proceeding.

- The role of dietary fibre intake or fibre supplementation in symptoms of depression and anxiety [published 23 September 2021] [CRD42021274898]. The review was judged **not to be similar**
- The effect of dietary fibre on gut microbiota composition in healthy adults: a systematic review and meta-analysis [published 10 December 2016] [CRD42016053101]. The review was judged **not to be similar**
- Exploring patterns in food intake and diet, nutritional status and water quality among people with Chronic Kidney diseases in Sri Lanka [published 10 May 2022] [CRD42022323219]. The review was judged **not to be similar**
- High dietary fiber intake in children and growth, bowel function and iron status [published 29 November 2021] [CRD42021288211]. The review was judged **not to be similar**
- What is the relationship between dietary fibre intake and markers of glucose control in pregnant women with diabetes? [published 19 July 2022] [CRD42022347344]. The review was judged **not to be similar**
- Systematic review of observational studies in humans on dietary fibre intake and prevention of asthma, rhinitis and lung function impairment [published 29 September 2021] [CRD42021275389]. The review was judged **not to be similar**
- Gut microbiome outcomes associated with dietary intake of cereal and grain foods: A systematic review [published 5 September 2018] [CRD42018107117]. The review was judged **not to be similar**
- Influence of socioeconomic and food accessibility factors on dietary behaviours, nutritional status and brain health outcomes among older adults. A systematic review [published 17 June 2024] [CRD42024542261]. The review was judged **not to be similar**
- Effect of dietary fibre intake on periodontal diseases- a systematic review of randomized controlled trials [published 13 November 2021] [CRD42021284997]. The review was judged **not to be similar**
- Systematic review of characteristics of dietary fibre in clinical intervention trials of healthy humans in relation to appetite and energy intake [published 1 April 2015] [CRD42015015336]. The review was judged **not to be similar**

TIMELINE OF THE REVIEW

Date of first submission to PROSPERO

This record has not been submitted.

Review timeline

Start date: 30 December 2025. End date: 28 February 2026.

Date of registration in PROSPERO

This record has not been published.

AVAILABILITY OF FULL PROTOCOL

Availability of full protocol

A full protocol has been written and uploaded to PROSPERO. The protocol will be made available after the review is completed.

SEARCHING AND SCREENING

Search for unpublished studies

Only published studies will be sought.

Main bibliographic databases that will be searched

The main databases to be searched are *PubMed* and *Scopus*.

Search language restrictions

The review will only include studies published in English.

Search date restrictions

Databases will be searched for articles published from 1 January 2006, there are no search end date restrictions.

Other methods of identifying studies

No other methods will be used.

Link to search strategy

A full search strategy is available in the full protocol as described in the *Availability of full protocol* section

Selection process

Studies will be screened by one person (or a machine) and checked by at least one other person (or machine).

Other relevant information about searching and screening

None

DATA COLLECTION PROCESS

Data extraction from published articles and reports

Data will be extracted by one person (or a machine) and checked by at least one other person (or machine).

Authors will not be contacted for further information.

Study risk of bias or quality assessment

Risk of bias will be assessed using: *Cochrane RoB-2* and *ROBINS-I*

Data will be assessed by one person (or a machine) and checked by at least one other person (or machine).

Additional information will **not** be sought from study investigators if required information is unclear or unavailable in the study publications/reports.

Reporting bias assessment

Risk of bias due to missing results will not be assessed

Certainty assessment

Certainty of findings will not be assessed

OUTCOMES TO BE ANALYSED

Main outcomes
if available: kidney function markers (eGFR, serum creatinine), inflammatory markers (CRP, TNFa, interleukins), uraemic toxins (pCS, IS), nutritional status (BMI, serum albumin)

Additional outcomes
There are no additional outcomes.

PLANNED DATA SYNTHESIS

Strategy for data synthesis
No formal data synthesis is planned - data will be described but not combined.

CURRENT REVIEW STAGE

Stage of the review at this submission

Review stage	Started	Completed
Pilot work	✓	✓
Formal searching/study identification	✓	
Screening search results against inclusion criteria	✓	
Data extraction or receipt of IPD		
Risk of bias/quality assessment		
Data synthesis		

Review status
The review is currently planned or ongoing.

Publication of review results
Results of the review will be published in English.

REVIEW AFFILIATION, FUNDING AND PEER REVIEW

Review team members
Ms Anna Gabriela Mojak (review guarantor and contact) ORCID: 0009-0002-7385-0973. Human Health Sciences Institute, University of Opole. Poland.

No conflict of interest declared.

Professor Monika Bronkowska. ORCID: 0000-0003-2960-6981. Human Health Sciences Institute, University of Opole. Poland.

No conflict of interest declared.

Named contact
Ms Anna Gabriela Mojak (132901@student.uni.opole.pl). ORCID: 0009-0002-7385-0973. Human Health Sciences Institute, University of Opole. Poland.

Review affiliation
Human Health Sciences Institute, University of Opole

Funding source
Review has no specific/external funding but is supported by guarantor/review team (non-commercial) institutions.

Additional information about funding
Supported as a part of doctoral research at the University of Opole

Peer review
There has been no peer review of this planned review.

ADDITIONAL INFORMATION

Review conflict of interest

Declared individual interests are recorded under team member details.. No additional interests are recorded for this review.

Medical Subject Headings

Classification; Dietary Fiber; Dietary Supplements; Humans; Inflammation; Kidney; Nutritional Status; Renal Insufficiency, Chronic; Uremic Toxins

PROSPERO version history

No preview available

Disclaimer

The content of this record displays the information provided by the review team. PROSPERO does not peer review registration records or endorse their content.

PROSPERO accepts and posts the information provided in good faith; responsibility for record content rests with the review team. The guarantor for this record has affirmed that the information provided is truthful and that they understand that deliberate provision of inaccurate information may be construed as scientific misconduct.

PROSPERO does not accept any liability for the content provided in this record or for its use. Readers use the information provided in this record at their own risk.

Any enquiries about the record should be referred to the named review contact
