# Supplementary material for: Dietary Fibre and Chronic Kidney Disease: A Systematic Review of Effects on Inflammation, Uraemic Toxins, Nutritional Status, Kidney Function, and Gut–Liver–Kidney Axis Mechanisms
Source: Nutrients. 2026 Apr 24;18(9):1341. doi: 10.3390/nu18091341 (PMC13165172; doi:10.3390/nu18091341)
Supplement: Supplementary file 1 [file nutrients-18-01341-s001.zip › RoB_presentation.pdf]

RoB 2.0	Domain 1	Domain 2	Domain 3	Domain 4	Domain 5	overall RoB
**Headley** (2025)	low	some concerns	some concerns	low	some concerns	some concerns
**Azevedo** (2020)	some concerns	some concerns	high risk	low	some concerns	high risk
**Kemp** (2021)	low	some concerns	high risk	low	low	high risk
**Esgalhado** (2018)	some concerns	low	low	low	some concerns	some concerns
**de Paiva** (2020)	some concerns	low	low	low	some concerns	some concerns
**Laffin** (2019)	some concerns	some concerns	low	low	some concerns	some concerns
**Khosroshahi** (2019)	low	low	low	low	low	low
**Khosroshahi** (2018)	low	low	low	low	low	low
**Poesen** (2016)	low	low	low	low	low	low
**Cui** (2024)	low	low	low	low	low	low
**Pajk** (2025)	some concerns	low	low	low	some concerns	some concerns
**Kwon** (2024)	low	some concerns	low	low	low	some concerns
**Jarupala** (2023)	some concerns	some concerns	low	low	some concerns	some concerns
**Elamin** (2017)	some concerns	high risk	some concerns	some concerns	some concerns	high risk
**de Andrade** (2021)	low	some concerns	low	low	low	some concerns
**Chang** (2023)	low	some concerns	some concerns	low	low	some concerns
**Ebrahim** (2022)	low	some concerns	some concerns	low	some concerns	some concerns
**Ramos** (2019)	low	low	low	low	low	low
**Meksawan** (2016)	some concerns	low	some concerns	low	some concerns	some concerns
**Li** (2020)	low	some concerns	some concerns	low	low	some concerns
**Li** (2022)	some concerns	some concerns	high risk	low	some concerns	high risk
**Tayebi Khosroshahi** (2016)	some concerns	low	low	low	some concerns	some concerns
**Xie** (2015)	some concerns	some concerns	low	low	low	some concerns

ROBINS-I	Domain 1	Domain 2	Domain 3	Domain 4	Domain 5	Domain 6	Domain 7	overall RoB
**Khalid** (2021)	serious	moderate	moderate	low	moderate	low	serious	serious
**Farman** (2020)	critical!	serious	low	serious	low	moderate	serious	critical!
**Salmean** (2013)	serious	low	low	moderate	low	moderate	moderate	serious
**Salmean** (2015)	serious	moderate	low	low	low	moderate	moderate	serious
**Hill** (2020)	serious	serious	low	serious	serious	moderate	serious	serious
**Ebersolt** (2022)	moderate	moderate	low	low	moderate	low	moderate	moderate
**Lai** (2019)	serious	moderate	low	low	low	low	low	serious
**Gao** (2020)	serious	moderate	low	moderate	low	low	moderate	serious
**Sohn** (2024)	serious	moderate	low	moderate	low	low	moderate	serious

JBI CSS	Domain 1	Domain 2	Domain 3	Domain 4	Domain 5	Domain 6	Domain 7	Domain 8	overall RoB
**Pan** (2025)	**Y**	**Y**	**PY**	**Y**	**Y**	**PY**	**Y**	**PY**	**moderate**
**Udomkarnjananun** (2025)	**Y**	**Y**	**PY**	**Y**	**PY**	**Y**	**Y**	**PY**	**serious**
**Hosseini (2020)**	**Y**	**Y**	**Y**	**Y**	**Y**	**Y**	**Y**	**Y**	**low to moderate**
**Xu** (2015)	**Y**	**Y**	**Y**	**Y**	**Y**	**Y**	**Y**	**Y**	low to moderate
**Lu** (2017)	**Y**	**Y**	**Y**	**Y**	**Y**	**Y**	**Y**	**Y**	**low to moderate**
**Kaesler** (2021)	**Y**	**Y**	**PY**	**Y**	**Y**	**Y**	**Y**	**Y**	**moderate**
**Xu** (2014)	**Y**	**Y**	**Y**	**Y**	**Y**	**Y**	**Y**	**Y**	low to moderate
**Rossi** (2015)	**Y**	**Y**	**Y**	**Y**	**Y**	**Y**	**Y**	**Y**	**low to moderate**
**El Amouri** (2021)	**Y**	**Y**	**Y**	**Y**	**Y**	**Y**	**Y**	**Y**	**low to moderate**
**Krishnamurthy** (2012)	**Y**	**Y**	**PY**	**Y**	**Y**	**Y**	**Y**	**Y**	**moderate**
**Ramos**	**Y**	**Y**	**Y**	**Y**	**PY**	**Y**	**Y**	**Y**	**moderate**

JBI CSS	Selection	Comparability	Outcome	overall RoB
**El Amouri** (2021)	**3/4 stars**	**2/2 stars**	**3/3 stars**	**8/9 stars**
**Xu** (2016)	**4/4 stars**	**2/2 stars**	**3/3 stars**	**9/9 stars**
